# Pharmaceutical Public Health: A Mixed-Methods Study Exploring Pharmacy Professionals’ Advanced Roles in Public Health, Including the Barriers and Enablers †

**DOI:** 10.3390/pharmacy13020037

**Published:** 2025-03-01

**Authors:** Diane Ashiru-Oredope, Roeann Osman, Adeola H. Ayeni, Eleanor J. Harvey, Maria Nasim, Emma Wright, Christina Narh, Uju Okereke, Tasmin Harrison, Christopher Garland, Cecilia Pyper, Andrew Evans, Marion Bennie

**Affiliations:** 1 Clinical and Public Health Group, UK Health Security Agency, London E14 4PU, UK; roeann.osman.20@ucl.ac.uk (R.O.); eleanor.harvey@nbt.nhs.uk (E.J.H.); maria.nasim89@gmail.com (M.N.); 2 School of Pharmacy, University of Nottingham, Nottingham NG7 2RD, UK; 3 Public Health, Teeside University, Middlesborough TS1 3BX, UK; adeola.ayeni@cntw.nhs.uk; 4 Public Health Team, Northumberland County Council, Newcastle NE61 2EF, UK; emma.wright@ukhsa.gov.uk; 5 Pharmacy Department, Barts Health NHS Trust, London E1 2ES, UK; c.narh@nhs.net; 6 NHS England East Midlands Team, NHS England, Birmingham LE3 8RA, UK; ujuokereke@warwickshire.gov.uk; 7 Public Health Action Support Team (PHAST), London SL9 7QE, UK; tasmin.harrison@phast.org.uk (T.H.); cecilia.pyper@phast.org.uk (C.P.); 8 Pharmaceutical Directorate, Department of Health, Belfast BT4 3SQ, UK; christopher.garland@health-ni.gov.uk; 9 Health and Social Services Group, Welsh Government, Cardiff CF10 3NQ, UK; andrew.evans@gov.wales; 10 Public Health Scotland, Edinburgh EH12 9EB, UK; marion.bennie@phs.scot

**Keywords:** pharmacy, pharmacoequity, health inequalities, emergency preparedness resilience and response, community pharmacy, policy, pharmaceutical public health, CORE20PLUS

## Abstract

Background: In the UK and globally, pharmacy professionals (pharmacists and pharmacy technicians) contribute to the delivery of local and national public or population health interventions. The existing literature on pharmaceutical public health predominantly focuses on micro-level activities, primarily describing community pharmacies delivering public health interventions to individuals. There is little-known evidence on pharmacy professionals’ involvement in delivering public health interventions at meso- (e.g., organisational) and macro (national/policy) levels, nor to what extent pharmacy professionals have specialist/advanced roles within public health practice. This study specifically explored pharmacy professionals’ specialist/advanced roles within public health as well as the opportunities and barriers to career development. The analyses of this mixed-methods study makes a series of important recommendations for future action. Methods: This study included two independent cross-sectional electronic surveys for pharmacy professionals and public health professionals, a call for evidence, and two workshops to develop recommendations. Results: Pharmacy professionals (n = 128) and public health professionals (n = 54) across the UK participated in the surveys. Most of the Pharmacy Professionals respondents were female (70%), pharmacists (85%), working in primary (33%) or secondary (25%) care settings, mainly based in England (75%), and most (63%) lacked formal public health qualifications although they were involved in a diverse range of public health interventions. The public health professionals were mostly females (67%), practicing in England (58%). Both professional groups identified opportunities and barriers to pharmacy professionals’ involvement in public health. Almost half of the public health professionals respondents (44%) stated that they had a pharmacy professional working as part of their current public health teams. Eighty-seven percent of public health professional respondents (45/52) agreed that having pharmacists or pharmacy technicians specialising in public health would be beneficial or very beneficial. Most of the documents, reports, and case histories provided through the call for evidence were unpublished. The workshops generated 94 recommendation statements, highlighting collaboration and the need to acknowledge pharmacy professionals’ contributions to public health. Conclusion: The recommendations for strategic action at meso- and macro-levels included three main themes: adopting a national strategic approach to pharmaceutical public health, including improving commissioning; formalising pharmaceutical public health workforce development; and promoting further evidence-based pharmaceutical public health research and development.

## 1. Introduction

**Figure 1:**
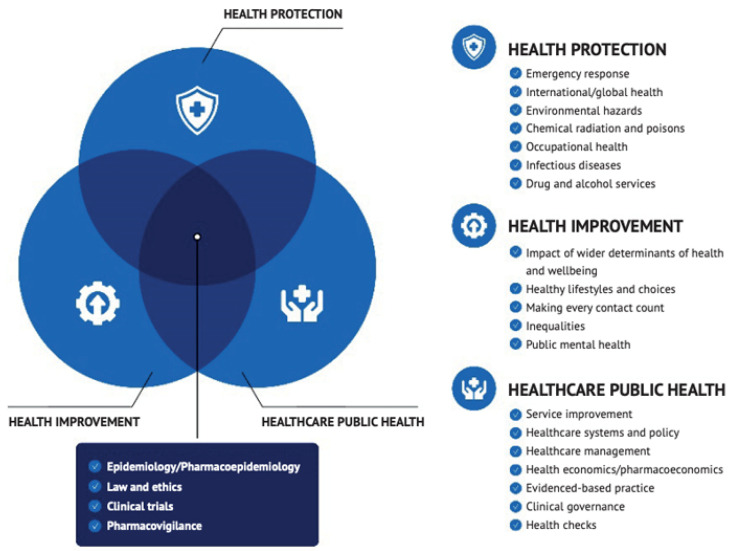
Domains of public health (reproduced with permission from Ashiru-Oredope, Population and public health, Pharmacy Magazine, https://www.pharmacymagazine.co.uk/cpd-modules/population-and-public-health) (accessed on 17 January 2025).

Pharmacy professionals (pharmacists and pharmacy technicians) have an increasing role in protecting as well as improving the health and wellbeing of populations and communities. Currently, pharmacy professionals work across the three core domains of public health practice [1]: health improvement, health protection, and healthcare public health (Figure 1 and Box 1), regarding population care beyond individual health outcomes. Pharmaceutical public health (PPH) was first defined in the literature in 2000 as “the application of pharmaceutical knowledge, skills and resources to the science and art of preventing disease, prolonging life, promoting, protecting, and improving health for all through organised efforts of society” [2]. At the time, Walker recognised that “Pharmaceutical Public Health is a real value-added role that the profession has, to date, chosen not to exploit” [2].

Box 1 **Definitions of public health, and domains of public health and population health [1].**

- Public health is defined as “the art and science of preventing disease, prolonging life and promoting health through the organized efforts of society”.
- Health protection is the protection of individuals, groups, and populations through the effective collaboration of experts in identifying, preventing, and mitigating the impacts of infectious diseases and of environmental, chemical, and radiological threats.
- Healthcare public health is concerned with the application of population sciences to the design, organisation, and delivery of healthcare services, with the aim of improving population health.
- Health improvement is concerned with assessment of population health needs, and commissioning and evaluating health programmes and initiatives to promote healthy behaviours. These include improving nutrition, physical activity, sexual health, substance use, disease prevention, and the importance of vaccinations.
- Population health is an approach aimed at improving the health of an entire population. It focuses on improving the physical and mental health outcomes and wellbeing of people, whilst reducing health inequalities within and across a defined population. It includes actions to reduce the occurrence of ill health, including addressing wider determinants of health, and requires working with communities and partner agencies.

Walker’s observation is still reflected more than 20 years later, with the relatively narrow focus of the literature on PPH. Mulvale and colleagues organised contextual factors as an interrelated set of policy (macro), organisational (meso), team (micro), and individual factors [3]. Over the years, the available literature on the pharmacy professions’ public health function has predominantly focused on the contribution of community pharmacies and pharmacists delivering public health interventions to individuals, through micro-level activities. Examples of these micro-level services include health promotion activities, administering vaccines, educating patients about vaccinations, conducting health screens targeting blood pressure, cholesterol, and weight, or secondary prevention measures through medicinal management and prescribing advice, rather than on population health or broader public health interventions [4]. Community pharmacies are frequently located in some of the most deprived and challenging communities, providing daily contact for individuals seeking ad hoc and unplanned health advice alongside a collection of prescribed medicines or purchasing over-the-counter health-related products [5,6]. In England, there are over 1.2 million daily health-related visits to community pharmacies. This presents an important opportunity to support behavioural change at an individual, micro level. However, there are also opportunities for pharmacy professionals across all sectors to address wider societal aspects of public health at meso- (network or systems through which micro systems interact, usually, e.g., community/organisational-focused) and macro (policy/population-focused) levels [3,5,6]. Examples of meso-level services include hosting health fairs or workshops to promote preventative health measures, working with community groups to address specific health disparities, designing primary or secondary disease-prevention programmes such as weight management, managing chronic disease initiatives across health systems, and advocating or facilitating programmes for low-cost or free medications. At the macro level, examples include advocating for legislation to improve access to medicines or pharmacy services, leading or contributing to policy development on vaccination mandates, conducting studies to assess health outcomes, addressing disparities in pharmaceutical access and outcomes, developing guidelines or educational materials for large populations, or promoting public health in national health departments such as ministries of health, public health bodies (such as UK Health Security Agency, Public Health Wales, and Public Health Scotland (in the UK), Centres for Disease Control such as Africa CDC, US CDC, WHO, or Non-Governmental Organisations.

In the UK, key population health policies include the following: in England, the NHS Long-Term Plan includes the development of integrated care systems (ICSs), integrated care boards (ICBs), and primary care networks (PCNs); in Scotland, the Public Health Priorities for (2018); in Northern Ireland, ‘Making Life Better—Strategic Framework for Public Health (2012-23); and in Wales, A Healthier Wales: Long-Term Plan for Health and Social Care [7,8,9,10]. These policies have the clear aim of improving the public health and wellbeing of the UK population by bringing together multi-professional groups, including pharmacy professionals, to coordinate care better.

**Figure 2:**
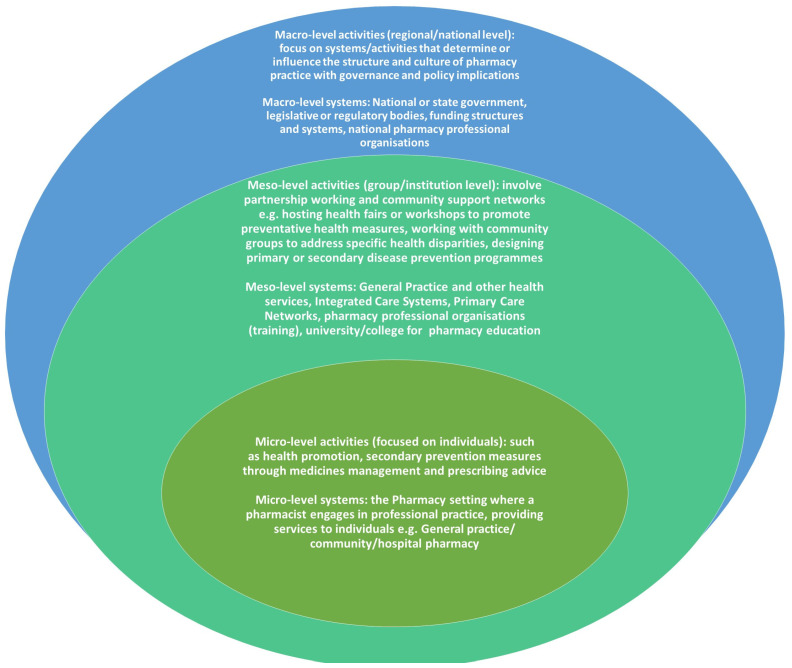
Micro-, meso-, and macro-level public health activities of pharmacy professionals.

An evidence gap currently exists that describes the involvement of pharmacy professionals working at the meso- and macro levels. The meso level involves partnership working and involvement in community support networks (group/institution level); the macro level involves influencing and working within local, regional, and national governments on complex agendas such as tackling health inequalities and implementing health policies (Figure 2). Involvement at these broader levels requires advanced public health skills in addition to pharmacy specialty skills.

An evidence review (commissioned by the UK Chief Pharmaceutical Officers in 2020) aimed to address the UK national policy direction publications [7,8,9,10] and pharmacy professionals’ specialist public health contributions. In addition to a rapid systematic review of evidence (unpublished), this evaluation study aimed to assess the barriers and opportunities to contributions at the meso- and macro levels of public health, and to make recommendations for future action. Knowledge of the barriers and opportunities that pharmacy professionals face in public health can serve to advance and optimise their contributions to national policy. The overall aim was to provide evidence that could contribute as part of the national policy directions on expanding pharmacy professionals’ role in population and public health.

## 2. Methods

### 2.1. Survey of Pharmacy and Public Health Professionals—Overview and Data Analysis

Two independent cross-sectional electronic surveys were developed, piloted, and deployed to pharmacy professionals and public health specialists via email and social media cascades. The surveys explored the extent to which pharmacy professionals are involved in public health roles, including opportunities and barriers encountered.

A combination of purposive and convenience sampling was employed to identify pharmacy and public health professionals in all the four UK nations (England, Scotland, Wales, and Northern Ireland).

The surveys (Supplementary Materials S1 and S2) were developed by pharmacist researchers with experience and advanced skills in public health and a public health professional (authors—DAO, UO, EW, CN, RO, MB, AE, CP, and CG), after an extensive literature review and regular meetings to assess the survey questions. The surveys were pilot-tested and reviewed by the study researchers and independent researchers, and the surveys were revised following feedback from reviewers. Independent researchers outside the core project team of researchers and participants with similar demographics to intended participants (8 pharmacists and 14 public health professionals identified by convenience sampling) were then asked to pilot test the survey to ensure clarity; revisions were made based on feedback.

The links to the surveys were disseminated via social media platforms (including LinkedIn, Twitter (now X), and WhatsApp, as well as via email to universities with public health courses and public health offices at acute trusts. In addition, they were disseminated during relevant teleconference calls and direct contact with professional colleagues. The survey for public health professionals was also disseminated by the Faculty of Public Health and the Association of Directors of Public Health through email and newsletter cascade routes.

The questions comprised mainly closed-ended multiple choice questions, alongside questions that allowed for open-ended responses inviting additional insights. Respondents were provided with opportunity to append links to previously published relevant work they were aware of.

Participation in both surveys was voluntary and consent to participate was collected as part of the questionnaire. Data responses were collected anonymously, although survey respondents could voluntarily provide their name and email address via a separate link at the end of the survey, with personal information disaggregated from their survey responses. This gave these individuals the opportunity to join a network of pharmacists with interest and experience in public and population health.

Survey data were imported into Microsoft Excel for analysis. The response rate was not calculated, since information was not available on the number of unique individuals who viewed or initiated the survey. Descriptive statistics were used to summarise the quantitative data collected. Missing data were excluded from analysis. Open-ended free text responses were analysed using inductive content analysis.

All data were stored securely in line with the General Data Protection Regulation 2016/679.

#### 2.1.1. Survey of Pharmacy Professionals

The intended audience for the pharmacy survey were pharmacy professionals (pharmacists and pharmacy technicians) across all four UK nations. The survey included 32 questions and remained open for responses over a 4-week period (25 June 2021 till 27 July 2021).

The objectives of the pharmacy professionals survey were to:

▪ explore the number of pharmacy professionals who have experience in leading public/population health projects as well as examples of the public health projects they had been involved in. In addition, participants were asked how the projects were disseminated. The survey included a comment box to provide further details about the projects and how to access the published information about the projects.
▪ explore their completion of, or ongoing additional public/population health qualification or training. The public health qualifications assessed included academic degrees, certifications, and professional credentials recognised for equipping individuals with the knowledge and skills needed to tackle public health challenges. The options covered undergraduate degrees, as well as postgraduate modules, certificates, diplomas, or degrees in public health, global health, health services, or specialised areas within public health, such as epidemiology, health protection, and health promotion. The option to include other qualifications was provided.
▪ explore the context in which pharmacists are currently involved in public/population health-related roles (excluding nationally commissioned public health services through community pharmacy).
▪ understand the barriers and enablers/opportunities associated with pharmacy professionals undertaking public/population health roles.

#### 2.1.2. Survey of Public Health Professionals

The intended audience for the public health professionals survey were public health specialists, registrars, and public health practitioners. Public health professionals refers to a core group of public health personnel that have undergone professional training and/or registration with professional bodies in public health and could be from either health or another background. Pharmacy professionals are not recognised as public health professionals without additional public health qualifications or credentialling.

The survey included 35 questions and remained open for responses over a 6-week period (15 September till 27 October 2021). The additional two weeks for the public health professionals’ survey (compared to the pharmacy professionals’ survey) was required for professional bodies to cascade the information through their newsletters.

The objectives were to seek the views of public health professionals on:

○ the potential functions of public health that can benefit from pharmacy professionals’ unique expertise including access to care and prevention services, as well as pharmacotherapy, pharmacoepidemiology, and economics;
○ the contributions of pharmacy professionals to public/population health (in addition to traditionally/nationally commissioned community pharmacy services) that they were aware of in the four UK nations;
○ their experience of working with pharmacy professionals;
○ examples of how correct and efficient use of medicines currently or previously had arisen as an area of challenge or consideration;
○ their perceptions of the benefits, barriers, and opportunities of pharmacists/pharmacy technicians specialising in public health and areas of public/population health they felt would benefit having individuals with pharmacy backgrounds working directly as part of the public health team.

The conduct and reporting of the study adhered to the Consensus-Based Checklist for Reporting of Survey Studies (CROSS). The completed CROSS checklist is available as supplementary information.

### 2.2. Call for Evidence

A call-for-evidence questionnaire (Supplementary Material S3) was sent by the Project Lead (DAO) on 25 June 2021 via email to key stakeholders who held senior pharmacy related positions in the UK for further cascade. The questionnaire was designed to identify any published or unpublished reports, documents, or case histories related to PPH. In addition, they were asked to provide examples of pharmacists in the UK who have received public health training or had experience working at a strategic level and influencing population health in the UK. To optimise responses, additional reminders were sent. And an extension until 16 July was sent out on 5 July 2021 (initial deadline).

The evidence received from published or unpublished reports, documents, or case histories was collated via the questionnaire and classified under one of 8 topic headings:

1. National Strategic Approach
2. Expanding Service Delivery within Community Pharmacy
3. Expanding Service Delivery beyond Community Pharmacy
4. Embedding Optimisation of Medicines at a Population Health Level
5. Emergency Preparedness, Resilience and Response
6. Integration of Pharmacy to Better Support Public Health Protection and Improvement Goals
7. Public Health Skills and Training
8. Mitigating Health Inequalities

Findings were shared via two workshops, each with a selected group of stakeholders, to promote discussion and help clarify questionnaire responses.

### 2.3. Workshops

Two workshops were held with key stakeholders on 16 July 2021 and 2 September 2021. The stakeholders selected through targeted approach were pharmacy professionals in public/population health roles in local, regional, or national organisations across the 4 UK nations. The first workshop included the participation of key stakeholders in a presentation and discussion regarding the mixed-methods review, a literature review, key findings and interim survey, and call-for-evidence findings.

Ahead of the second workshop, a slide-set summary of the literature review (unpublished), the interim results of the two surveys, and a call for evidence were sent to invitees, with a request via a Slido tool (Supplementary Material S4) for them to suggest recommendations on how to steer national changes to improve pharmacy professionals’ contributions to public health across the four UK nations, in addition to what already exists as commissioned community pharmacy services. These recommendations were further discussed at the workshop (Supplementary Box S1 includes the workshop agenda) and each participant was asked to share which one recommendation they deemed most important regarding the involvement of pharmacy professionals in PPH.

### 2.4. Ethics Approval and Consent

Ethical approval was not required according to the NHS Health Research Authority tool; the surveys aimed to evaluate through exploration the specialist contributions of pharmacy professionals to national policies/direction on public health. Consent was sought from all participants and the anonymity of contributions ensured. In addition, an internal review by the UKHSA Research Support and Governance Office (RSGO) provided a response by email that stated “this study fell outside the remit for ethical review. This decision was made by virtue of this work sitting more closely within the remits of a service evaluation, and that since the work exclusively involved participants by virtue of their professional roles, and as the study was not asking questions that collect personal or identifiable information, or that could cause participants anxiety, an ethical review of the project was not required”.

## 3. Results

### 3.1. Survey of Pharmacy and Public Health Professionals

#### 3.1.1. Survey of Pharmacy Professionals

##### Demographics

A total of 128 pharmacy professionals (85% pharmacists) responded from the four UK nations (n = 96 (England), n = 13 (Scotland), n = 9 (Wales), n = 9 (Northern Ireland), and n = 1 (stated Great Britain)) (Table 1). Respondents were predominantly female (70%; 90/128). Most had a White British background (48%; 62/128), 14% (19/128) were Asian or Asian British, and 12% (14/128) were Black or Black British. Within England, most respondents worked in the South East of England (20%; 20/96)), while 19% (18/96), 17% (17/96), and 11% (11/96) worked in Midlands, London, and the North East and Yorkshire, respectively. Regions that were represented in lower proportions included the South West, North West, and East of England.

**Figure 3:**
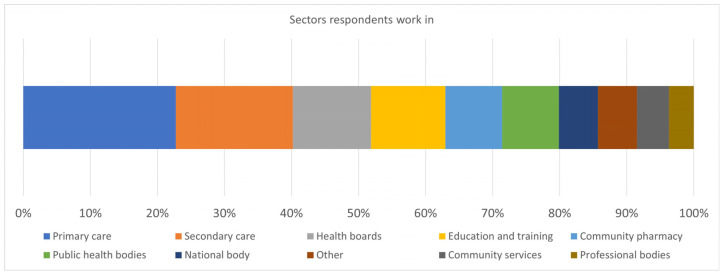
The sectors pharmacy respondents work in.

Respondents worked in a range of sectors including primary care, secondary care, health boards, public health bodies, community pharmacies, local authority, and health and justice settings (Figure 3). Many respondents (34%; 43/128) worked in the primary-care setting and 26% (33/128) in the secondary-care setting. Thirteen per cent (16/128) stated they worked across both the community setting and public health bodies. Eighty-five per cent (109/128) of survey respondents stated their role was a pharmacist, while 15% (19/128) stated pharmacy technician. No trainees (pharmacists or pharmacy technicians) responded to the survey.

##### Public Health Qualifications and Motivation

Most of the respondents (63%, 80/128) had no formal public health qualification (i.e., academic degrees, certifications, or professional credentials recognised for equipping individuals with the knowledge and skills needed to tackle public health challenges), 27% (34/128) had formal public health qualifications, and the remaining 10% (14/128) had a qualification in progress. For those who held a public health qualification, the year of qualification ranged from 1 to 49 years ago. One respondent was a Fellow of the Faculty of Public Health, and another was a member of the Royal Society of Public Health. In response to the question “How long have you been using your public health qualification or skills within role(s)?”, over half of those that responded to the question (55%, 16/29) answered they had been using their public health qualifications or skills within their roles for five or more years, 21% (6/29) had not used their qualification nor skills, and 14% (4/29) and 10% (3/29) had used their qualifications or skills for three to four years and one to two years, respectively.

The public health education modules most often completed by pharmacy professionals (most commonly as part of a Master in Public Health qualification) were health improvement/promotion (12%, 15/128), epidemiology (12%, 15/128), health policy (11%, 14/128), infectious and tropical diseases (10%, 13/128), health services (10%, 13/128), health systems (10%, 13/128), global health/global health policy (10%, 13/128), and health protection (9%, 11/128).

When respondents were asked what best described their motivation for undertaking an additional public/population health qualification(s), 31% (17/55) selected an ambition to work in public health as a pharmacy professional, and 29% (16/55) selected an ambition to work in public health as an alternative career to pharmacy. About a third (27%, 15/55) also selected that it was completed for general interest, 7% (4/55) and 4% (4/55) of the respondents stated that their motivation was based on recommendations received and qualifications required for their roles, respectively, while 2% (1/55) of respondents did not identify a specific motivation.

##### Public Health Experience

**Figure 4:**
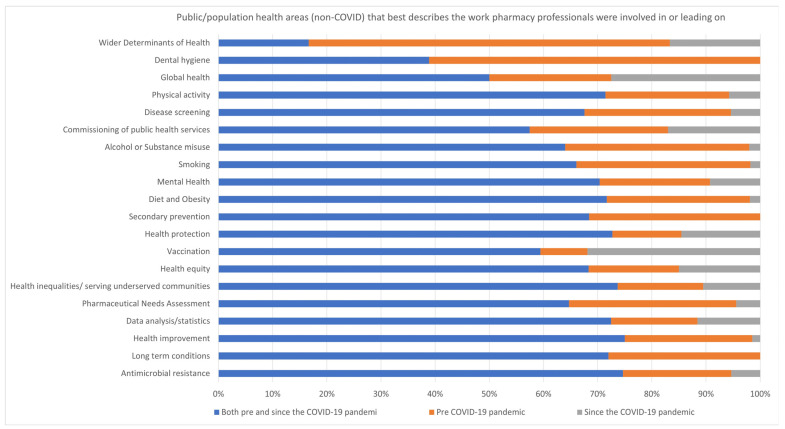
Public/population health areas (non-COVID-19) that best describe the work pharmacy professionals were involved in or leading on.

Within public health, pharmacy professionals were involved in various non-COVID-19-related public health areas, as shown in Figure 4. Over 70% of respondents were involved in antimicrobial stewardship activities to tackle antimicrobial resistance, managing long-term conditions, health improvement, and data analysis/statistics before and since the COVID-19 pandemic. The proportion of respondents that were engaged in the development of Pharmaceutical Need Assessments (PNA)** was 31% (21/68) before, and 4% (3/68) since the pandemic; for health inequalities, this was 16% (9/57) pre and 10% (6/57) since the pandemic.

Pharmacy professional respondents had conducted diverse public health projects. Approximately half (54%; 69/128) of pharmacy professionals had not shared project findings beyond their organisation; 25% (32/128) had their project findings disseminated during conference presentations and abstracts, 29% (28/128) via guidance, protocols or Patient Group Directions (PGDs) and 16% (20/128) as peer-review publications. Ten percent (13/1128) and nine percent (12/128) of respondents had their findings published in non-peer-reviewed publications or as blogs and online reports, respectively.

** “Section 128A of the National Health Service Act 2006 (NHS Act 2006) requires each health and wellbeing board to assess the need for pharmaceutical services in its area and to publish a statement of its assessment. Termed a ‘pharmaceutical needs assessment’”.

##### Barriers and Opportunities

Most respondents to the question on barriers (71%; 89/126) believed there are barriers for pharmacy professionals to engage in public and population health, 23% (29/126) of the respondents believed there might be barriers for pharmacy professionals to public and population health engagement and only 6% (8/126) did not believe barriers exist for pharmacy professionals to get involved in public and population health.

Eight key themes were identified from the barriers provided by respondents (Table 2 and Table S1). The highest number of comments mentioned as a barrier to PPH related to limited career opportunities or no defined career pathway.

Most respondents (80%; 102/127) believed opportunities exist for pharmacy professionals to engage in public and population health. Six themes were identified as opportunities for pharmacy professionals to engage in public or population health (Table 3 and Table S2). The most popular theme was that pharmacy professionals are well placed to make a public health impact.

There were specific barriers and opportunities highlighted by pharmacy technician respondents (Supplementary Boxes S2 and S3). The main barrier reported by pharmacy technicians was an underuse of skills. Technicians also remarked on a lack of professional accreditation which resulted in a gap in service provision.

#### 3.1.2. Survey of Public Health Professionals

##### Demographics

A total of 54 public health professionals participated in the survey; 67% (36/54) were female, 30% (16/54) male, and 3% (2/54) preferred not to say (Table 4). Most respondents (59%, 32/54) were practising in England, 28 (15/54) in Scotland, and 13% (7/54) in Wales. Participants worked in a range of roles: 19% (10/54) were public health consultants, 9% (5/54) directors of public health, 11% (6/54) public health registrar ST4–5, and 13% (7/54) public health registrar ST1–3.

Almost half of the public health participants (44%, 24/54) reported they had pharmacy professionals as part of their current public health team. Fifty-two per cent (27/52) stated that they were aware of a pharmacy professional who was also a public health professional; example of roles included public health partnerships and improvement leads, chief pharmacist for Public Health Scotland, pharmacists guiding the analysis of national prescribing data and production of official statistics and ad hoc reports, and lead antimicrobial resistance pharmacists.

Thirty-one percent (10/32) of respondents to the question on providing placement for funded pharmacy professionals agreed that their organisation would be willing to provide a placement to a funded pharmacy professional to undertake a secondment or fellowship in public health, whereas nineteen percent (6/32) of respondents did not agree to provide a placement. A third (30%; 16/54) of respondents stated that their organisation may possibly provide a placement.

For the question, “Thinking about 12 months pre COVID-19 and within your public health role, did the correct and efficient use of medicines come up as an area of challenge or consideration?”, half of respondents (50%; 27/54) stated yes.

In England, more than half of the respondents (57%) reported that their public health teams had contributed to a Pharmaceutical Needs Assessment (PNA) in the previous 5 years. Of which, 47% stated that a pharmacy professional was part of the public health team and 40% did not know. In Scotland, Wales, and Northern Ireland, the survey captured the involvement of pharmacy professionals in the public health reports or delivery strategies, with a variable response across nations in the engagement of pharmacy professionals in reports.

Respondents were also asked what level of experience or qualification they believed was required for a pharmacy professional who wants to focus on medicines or pharmacy-related public health activities. Almost half (45%; 13/29) specified Masters in Public Health, and two specified credentials at a consultant level by a public health or pharmacy professional body (Box 2).

Box 2 **Examples of comments on the level of experience or qualification required for a pharmacy professional who wants to focus on medicines or pharmacy-related public health activities.**

- *“Bachelors degree standard public health qualification or masters modules in public health pertaining to determinants of health, inequalities, health economics (not necessarily the whole masters but perhaps a certain count of credits across key modules)”.*
- *“I think this would depend on what they were being asked to do however probably working towards masters in public health”.*
- *“MPH and working toward UKPHR if not on formal training scheme”.*
- *“MSc Pharmacy and MPH as minimum also at Royal Pharmaceutical Society consultant pharmacist ready status”.*
- *“Postgraduate qualification incorporating at least one module on public health or a public health related area. There are certain vocational aspects in healthcare where things can be learned on the job, e.g., clinical practice, however in public health you need to know fundamentals and these are best taught in an academic context”.*

##### Benefits and Barriers to Specialist Roles for Pharmacists in Public Health

**Figure 5:**
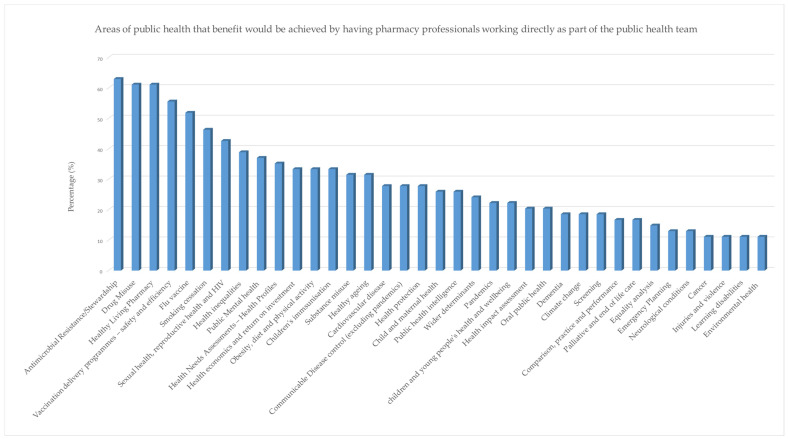
Areas of public health that benefit would be achieved by having pharmacy professionals working directly as part of the public health team.

Eighty-seven percent of public health professional respondents (45/52) agreed that having pharmacists or pharmacy technicians specialising in public health would be beneficial (37%) or very beneficial (50%), and thirteen percent agreed that it would be somewhat beneficial (Table 5 and Box 3). Respondents selected several areas of public health where they believed direct benefits would be realised and achieved by having direct involvement of pharmacy professionals. There were 15 areas that over a third of respondents selected as their top five areas (Figure 5), the top one being antimicrobial resistance/stewardship. Sixty percent of respondents (30/50) believed there are barriers for pharmacy professionals to get involved in public or population health, while twenty-six percent (13/50) believed that there are no barriers for pharmacy professionals. Very few participants (4%; 2/50) were unsure (Table S3).

Example quotes from respondents are provided in Box 3.

Box 3 **Examples of quotes from respondents on benefits of having pharmacy professionals as part of public health teams.**

- *“Pharmacy professionals’ knowledge of medicines and medicines use is second to none. This knowledge could be utilized to analyse medicines use data and the development of public health strategies and campaigns. There are several areas within public health where the expertise of a pharmacy professional would be beneficial including vaccination, antimicrobial stewardship, smoking cessation, weight loss management and substance misuse. Within health institutions multi-disciplinary working is fully embedded and the role of the pharmacy professional is appreciated but the same system of working has not been established within public health”.*
- *“They can bring knowledge of pharmacoeconomic, understanding of how pharmacy services are delivered in the community to the wider population, an independent prescriber would have the authority to help deliver health protection interventions, e.g., managing outbreaks”.*
- *“Pharmacies are ideally placed to provide local people with information to support behaviour change and start them on their journey to improved health and wellbeing. Health campaigns are promoted via pharmacy a lot, this model could be used to support behaviour change through, e.g., increased physical activity, signposting, raising the issue of PA or Healthy Weight, greenspace access etc”.*

The areas of public health options provided to respondents to select from were identified from the UK Faculty of Public Health, and Functions and Standards of a Public Health System—https://www.fph.org.uk/professional-development/good-public-health-practice/ (accessed on 17 September 2024) and https://www.fph.org.uk/media/3031/fph_systems_and_function-final-v2.pdf (accessed on 17 September 2024).

### 3.2. Call for Evidence

Forty-five stakeholders responded to the call for evidence. Sixty-nine percent (n = 31) were from England, eighteen percent (n = 80) from Northern Ireland, nine percent (n = 4) from Scotland, and four percent (n = 2) from Wales.

**Figure 6:**
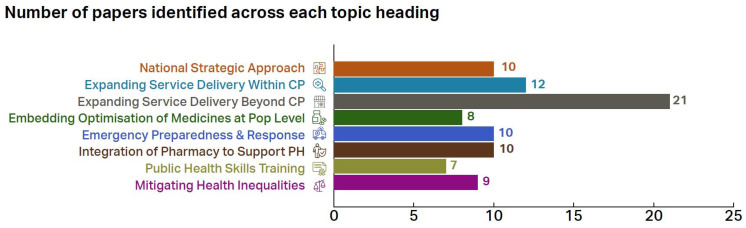
Number of documents, reports, and case histories identified and aligned with eight themes.

Most documents, reports, and case histories identified were unpublished, and the case histories varied in detail. The number of papers identified by the respondents across each of the eight topic headings are illustrated in Figure 6. Most papers (n = 21) related to expanding service beyond community pharmacy (CP), followed by expanding service delivery within CP (n = 12). Fewer papers were identified for public health skills training (n = 7), embedding the optimisation of medicines at population level (n = 8) and mitigating health inequalities (n = 9).

### 3.3. Workshops to Generate Recommendations

The first workshop was attended by 12 people and the second workshop by 42 people. Seven attendees from the first workshop also attended the second workshop.

Workshop attendees and contributors to the recommendations included pharmacy professionals from national public health organisations including Public Health England (PHE) (now part of UKHSA and OHID), NHS England (national teams), Public Health Scotland, and Public Health Wales, government health departments in England, Wales and Northern Ireland. Professional bodies like the Royal Pharmaceutical Society (RPS), Association of Pharmacy Technicians UK (APTUK), and Community Pharmacy England, which support pharmacy practice and public health delivery, were also represented. Additionally, pharmacy professionals based in regional entities like integrated care boards (ICBs) and regional teams in NHS England, academic and training institutions, universities, and Health Education England (HEE), which contribute to workforce development, as well as local authorities and integrated care trusts that deliver community-based health services attended.

From the two workshops, 94 recommendation statements were proposed and grouped under eight themes across meso- and macro levels (Table 6). Whilst the stakeholders were requested to focus recommendations on how to steer national changes to improve pharmacy professionals’ contribution to public health across the four UK nations, four of the recommendation statements included the following individual level actions that can be taken. These micro-level recommendations for individuals include:

- Championing the role of pharmacy professionals: individuals advocating for pharmacy professionals as key contributors to public health, both within and outside the pharmacy profession.
- Policy education and influence: Presenting research findings to pharmacy and public health bodies to educate decision-makers and inform future public health policy.
- Research opportunities and dissemination of knowledge: Exploring and participating in research opportunities to expand the role of pharmacy professionals in public health and actively engaging in the dissemination of research, audits, and projects to advance pharmaceutical public health (PPH).
- Medicine surveillance and intelligence: Contributing to the sharing of data for medicine surveillance and intelligence, facilitating the adoption of research and findings for improving public health.

For the full list of recommendation statements, see Supplementary Material S5.

## 4. Discussion

This exploratory study highlights the existing scope of pharmaceutical public health (PPH) and opportunities for pharmacy professionals to expand their contributions to public health. Griffiths et al. highlighted the importance of a diverse skill set in public health and noted that “different levels of skill and a wide range of contributions are needed if public health programmes are to make the most impact” [11]. The WHO-ASPHER Competency Framework for the Public Health Workforce provides comprehensive insights into the skill combinations for competent public health teams [12]. Pharmacy professionals in the UK, through their training, possess skills that align with all ten of the competency categories outlined by the WHO ASPHER framework. In addition, there appeared to be support from public health professionals regarding the impact and benefits of working in partnership with pharmacy professionals with advanced public health knowledge and skills, either directly or as part of a wider multidisciplinary public health team. As the health and care sectors face intense challenges exacerbated by a declining labour pool, innovative and diverse roles within the public health and pharmacy sectors can serve to unlock hidden potential [13].

Despite their potential, interest and contributions to public health interventions, most pharmacy professionals surveyed did not have formal public health qualifications and cited inadequate public health knowledge as a substantial barrier to further PPH involvement. Concurrently, public health professional respondents expected such qualifications to be available for pharmacy professionals, including public health masters courses or postgraduate modules in public health-related areas such as health economics and health inequalities. This highlights the need for dedicated PPH training and system-wide leadership to unlock pharmacy professionals’ contributions for population-level benefits.

The barriers and opportunities identified in this study can be categorised as macro-, meso-, or micro-level factors, following a socio-institutional framework which has been previously used to examine how healthcare professionals expand their scope of practice [3]. This framework recognises the opportunities that decision-makers have at each level in fostering effective collaboration within interprofessional care teams [14]. Macro factors include professional regulation, education, funding, and provider payment schemes [15,16,17,18,19,20,21,22]. Meso factors include organisational structure, rewards, and information systems. Micro factors include processes based on mutual trust, power-sharing that reflects knowledge and experience rather than titles as well as the knowledge and skills of professionals to determine their readiness to expand their role [23,24,25], and individual factors such as maturity in one’s profession and attitudes toward collaborative practices [26]. The delicate interactions between factors across all three levels shed light on the interconnectedness which is crucial for shaping and enabling collaborative practices within the ever-evolving public health landscape. Changes or barriers at one level (e.g., restrictions or limited national policies, boundaries of practice) can influence dynamics at other levels (e.g., limited organisation support at the meso level or reduced individual confidence at the micro level).

This study identified macro-level barriers such as limited pharmacy representation in public health domains and policy challenges, including those outlined in the NHS Long-Term Plan for England and those that emerged during the COVID-19 pandemic. However, the post-pandemic landscape presents clear opportunities to strengthen the role and contributions of pharmaceutical public health (PPH). Meso-level barriers identified by pharmacy professionals included organisational and structural barriers, a lack of training and support, inadequate professional recognition, and limited time and/or financial resources for pharmacy professionals to develop more advanced skills in public health. Meso-level opportunities as well as barriers were identified by at least half of the public health professional survey respondents, who had either previously or presently encountered pharmacy professionals working as members of public health teams or organisations. Additionally, over a third of respondents acknowledged 15 public health areas where pharmacy professionals across a range of sectors could directly add value. Collaborative public health efforts between healthcare professionals are highlighted within the UK Faculty of Public Health Functions and Standards of a Public Health System [27] and the Royal Society of Public Health’s “Unlocking the Potential of the Wider Public Health Workforce” report [28].

The micro-level barriers of day-to-day practice emphasised the limited career prospects and absence of defined career pathways for pharmacy professionals within the public health sector. This perceived lack of job opportunities also emphasises the broader issue of the scarce recognition of the core knowledge, skills, and qualifications pharmacy professionals bring to the public health sector. 

Recently, Todd and Ashiru-Oredope proposed that the definition of pharmaceutical public health should be updated to include health inequalities, suggesting the following definition: ‘*the application of pharmaceutical knowledge, skills and resources to the science and art of preventing disease, prolonging life, promoting, protecting, **improving health and reducing health inequalities** for all through organized efforts of society*’ [29]. Pharmacy professionals can make important contributions to meeting the objectives of frameworks that provide national approaches to support the reduction in health inequalities at meso and macro levels, such as the CORE20PLUS5 in England and Healthy People 2030 in the USA [30,31].

Consensus from the emerging barrier and opportunities themes of the surveys, a call for evidence, and recommendations from the workshops highlighted the contributions pharmacy professionals can make to public health at all levels. Also, the eight main themes identified from the proposed recommendations outline the potential multifaceted nature of pharmacy professionals’ roles within the public health setting. 

Further grouping the eight themes can result in the focus being on the macro level of national strategic approach and commissioning, the meso-level focus on training and workforce development, and the micro-level focus on individual pharmaceutical public health development and involvement in research. Whilst recent scoping reviews have proposed pharmaceutical public health competences for pharmacists [32,33], there is currently a gap in the literature for pharmacy technicians. A recent qualitative study including pharmacists from Australia, United Kingdom, Canada, and the United States of America concluded that development strategies are required to be more effective in integrating public health approaches into pharmacy professional practice and for them to be recognised for their public health-related roles [5]. The impact of meso- and macro-level strategies have recently been highlighted in the UK. The Allied Health Professions Federation, which is a professional body for 14 AHP professions including, e.g., physiotherapists, paramedics, dieticians, and radiographers, in 2024, published the impact of the UK Allied Health Professionals (AHP) Public Health Strategic Framework (2019–2024) [34]. Five years after the publication of the strategy, examples of national impact highlighted include public health pre-registration curricula guidance for AHPs by the professions’ UK regulatory body, the Council of Deans for Health, 100% of higher education institutes now including public health in AHP pre-registration education, published evidence reviews on the role of AHPs in public health, and the publication of high-impact case examples of AHPs leading or contributing to public health priorities, including across vision screening, health inequalities, fall prevention, and environmental sustainability.

A limitation of this study was the relatively low response rate. However, the consensus from the themes emerging from the findings of both surveys highlighted the contributions pharmacy professionals make towards public health despite the barriers. Future qualitative studies should be considered to investigate and identify how to best resource advanced PPH resources. Another limitation is that the study period occurred during the COVID-19 pandemic, which is likely to have influenced responses from pharmacy professionals. Whilst we attempted to mitigate against this by seeking details on non-COVID-19-related public health areas pharmacy professionals contributed to or led on pre and post COVID, this might have been impacted by recall bias. It is also possible that some pharmacy professionals commenced public health qualifications as a result of the COVID-19 pandemic, which we did not capture.

A key strength of this study is its use of the socio-institutional framework to consider the reported barriers and opportunities at the macro-, meso-, and micro levels. This framework has been applied in previous studies examining the role expansion of healthcare professionals, such as nurse practitioners’ roles in rural parts of Australia and pharmacists’ roles in public health in the USA. Insights into these interconnected factors from the framework helps identify strategies to address challenges and leverage opportunities. This can include employing strategies such as policy changes, enhancing organisational support, and strengthening individual competencies [35,36].

### Proposed Recommendations for Strategic Action

Based on the findings from this mixed-methods exploratory study (including the barriers and enablers highlighted by pharmacy and public health professionals through the surveys, the call for evidence, and recommendations proposed by workshop participants), we propose the following recommendations across macro- and meso levels:

Adopting a national strategic approach to pharmaceutical public health, including improving commissioning

- Leadership and Standards: Establish clearer regional and national leadership for pharmacy professionals working in population health and public health.
- Areas for Impact: Highlight areas where pharmacy professionals are well-placed to make a public health impact, such as their strategic position in the community.
- Regulatory Review: Review relevant regulations such as the Pharmaceutical Needs Assessment (PNA) regulations in UK, to highlight the role of pharmacy professionals in public health and include relevant data collection.
- Priority Setting: Identify and prioritise areas for public health contributions from pharmacy professionals at meso- and macro levels, particularly in underrepresented fields such as emergency preparedness and planning, health protection, medicine surveillance and interventions, the integration of primary, secondary and tertiary care, and addressing health inequalities. The latter is particularly important in areas with a high population of underserved communities.

Formalising pharmaceutical public health workforce development

- Career Pathways: Define professional standards and a clear career pathway for pharmaceutical public health (PPH) which enable pharmacy professionals to contribute to or lead within public health, including at strategic levels, whilst remaining within their profession. This should be supported by a robust competency framework and accreditation process.
- Curriculum Integration: Embed public health competences within undergraduate and postgraduate pharmacy programmes, such as foundation modules for undergraduates and public health components in clinical and prescribing courses.
- Specialisation and Recognition: Promote specialisation in PPH by enabling pharmacy professionals to pursue advanced credentials in public health; for example, through the Royal Pharmaceutical Society’s advanced and consultant pharmacist credentialing (in the UK). In the UK, joint recognition or registration with the pharmacy regulator (General Pharmaceutical Council) and Faculty of Public Health (or UKPHR) in a similar approach to medical professionals would also be a strong step to validating pharmacy professionals pursuing this avenue. For early-career pharmacy technicians and pharmacy support staff, integrate PPH into their respective training courses to emphasise the importance of pharmacy involvement in this area.
- Comprehensive Training: Develop training programmes for pharmacy professionals which include options to undertake public health activities, including health policy, wider determinants of health, and financial drivers of population health.
- Establishing a professional development network can provide peer support and foster continual workforce development.

Promoting evidence-based pharmaceutical public health research and development.

- Best Practices and Determination: Encourage the sharing of good PPH practice models and enhance the dissemination and adoption of research, audits and project findings by individuals to strengthen the PPH community.
- Research Funding: Create funding mechanisms for high-quality PPH research to increase engagement and collaboration in public health initiatives.

**Figure 7:**
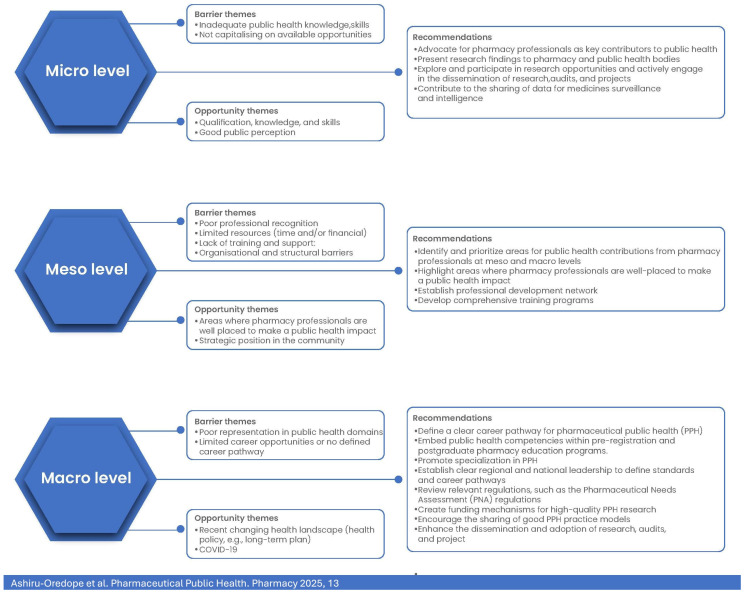
Summary of barrier themes and opportunity themes identified from surveys and recommendations from workshop participants at micro-, meso-, and macro levels.

These themes, alongside the micro-level recommendations, barriers, and opportunities identified from the surveys, are summarised in Figure 7.

## 5. Conclusions

This study highlights the evolving roles of pharmacy professionals in public health beyond micro-level interventions, usually through community pharmacy. It also emphasising the alignment of these roles with global competency frameworks and public health standards. Barriers, opportunities, and recommendations to increase PPH involvement were examined through a socio-institutional framework, considering, macro-, meso- and micro-level factors (summarised in Figure 6). To advance pharmacy professional involvement in PPH, this study recommends focusing on adopting a national strategic approach to pharmaceutical public health, including improving commissioning, formalising pharmaceutical public health workforce development, and promoting evidence based pharmaceutical public health research and development.

In the context of the rapidly evolving healthcare landscape, shaping a transformative future for PPH requires addressing interconnected factors across all levels. This approach will help identify innovative solutions to unlock the untapped potential of pharmacy professionals in public health, particularly at the meso- and macro levels. Future qualitative research should explore strategies to optimise advanced PPH resources for the benefit of populations.

## Figures and Tables

**Table 1:** Demographic characteristics of pharmacy-professional survey participants.

Demographic	Frequency (%)
Gender identity	
Female (including trans women)	90 (70)
Male (including trans men)	35 (27)
Prefer not to say	3 (3)
Ethnic group or background	
White—British	62 (48)
Asian or Asian British—Indian	13 (10)
White—Irish	12 (9)
Black or Black British—African	11 (9)
White—Any other White background	8 (6)
Prefer not to say	5 (4)
Asian or Asian British—Any other Asian background	3 (2)
Asian or Asian British—Pakistani	3 (2)
Mixed—Any other mixed background	3 (2)
Other Ethnic Groups—Chinese	3 (2)
Black or Black British—Caribbean	2 (2)
Black or Black British—Any other Black background	1 (1)
Mixed—White and Asian	1 (1)
Not stated	1 (1)
Country of work	
England region	96 (75)
South East	20 (21)
Midlands	18 (19)
London	17 (18)
North East and Yorkshire	11 (11)
National	9 (9)
East of England	8 (8)
North West	7 (7)
South West	6 (6)
Scotland	13 (10)
Northern Ireland	9 (7)
Wales	9 (7)
GB	1 (1)
Total	128 (100)

**Table 2:** Barriers to engaging in public and population health; themes identified and examples of comments given by respondents.

Barriers	Number of Comments (%)	Example Comments
Limited career opportunities or no defined career pathway	39 (19.7)	*“There is a lack of job opportunities for pharmacy professionals within public health teams themselves as there is a lack of recognition of the core knowledge and qualification that pharmacy professionals possess.”*
Poor professional recognition	34 (17.2)	*“As stated above and there appears to not be a good understanding from other healthcare professionals and the general public of the impact pharmacy professionals could have given the opportunity.”*
Limited resources (time and/or financial)	32 (16.2)	*“I’m not sure how you would go about it to fit in with an already highly pressured role in acute practice.”*
Lack of training and support	30 (15.2)	*“Lack of pharmacy specific formal training that can easily be accessed.”*
Inadequate public health knowledge, skills	21 (10.6)	*“I think the profession as a whole can struggle with the type of collaborative leadership that is required for this type of work.”* *“I think this is due the nature of our training historically—risk averse, looking for errors, lack of trust—not helpful for building collaborative relationships.”*
Organisational and structural barriers	19 (9.6)	*“There are opportunities, but we face barriers, specifically in terms of commissioning and public awareness, and for pharmacy technicians—legislation. We could provide information on prevention, healthy lifestyle choices, supply & administration of medicines, deprescribing etc”*
Not capitalising on available opportunities	12 (6.1)	*“There is certainly demand for public health professionals but the perceptions and mindset both of pharmacy professionals and the wider public health profession needs changing before individuals can really take advantage of current and future opportunities.”*
Poor representation in public health domains	11 (5.6)	*“Not many visible pharmacist leaders in this arena that can also influence delivery on the ground”*
Total	198	

**Table 3:** Opportunities to engage in public and population health: themes identified and examples of comments given by respondents.

Opportunities	Number of Comments (%)	Example Comments
Areas where pharmacy professionals are well placed to make a public health impact	45 (45.4)	*“The role of the pharmacist is evolving, and pharmacists can play a vital role in improving the health of populations through pharmacy services and the use of medicines and access to health information to empower patients.”*
Qualification, knowledge, and skills	26 (26.8)	*“Public health is currently a field dominated mostly by doctors, but pharmacists have a unique perspective and different knowledge base to offer to the field of public health.”*
Strategic position in the community	18 (18.6)	*“Very important area—pharmacies are embedded in the heart of our communities, see our population[s] more than any other health professional.”*
Recent changing health landscape (health policy, e.g., NHS Long-Term Plan)	4 (4.1)	*“There are many opportunities for community pharmacy professionals to be involved in public health interventions depending on capacity, training and commissioning of services, e.g., smoking cessation, sexual health, vaccination, substance misuse services, infection prevention and testing/treating and contributing to pathways for overweight and obesity”.* *“There are also opportunities for pharmacists employed by health boards to be involved in population health, e.g., prescribing/medicines management initiatives.”* *“The All Wales Therapeutics and Toxicology Centre has various working groups in which pharmacists can be involved in strategic medicines management/pharmaceutical public health which in some instances links to other sources of data to provide a broader perspective”*
COVID-19	3 (3.1)	*“Pharmacists played a central role in the excellent vaccine rollout in the UK, manufacturing of alcohol rubs, in providing advice on the administration and sourcing of medication to be used in COVID-19.”*
Good public perception	2 (2.1)	*“We are analytical, excellent communicators, efficient and brilliant decision makers. There are many opportunities for us to demonstrate this at a global, regional, and national level. The public and healthcare professionals trust our judgement and knowledge, so now is the perfect time to showcase our skills in public/ population health.”*
Total	97	

**Table 4:** Demographic characteristics of public health professional survey participants.

Category	Subcategory	Frequency (%)
Job Role of Respondent n = 54	Public health consultant	10 (19%)
	Public health registrar ST1–3	7 (13%)
	Public health registrar ST4–5	6 (11%)
	Public health practitioner	5 (9%)
	Director of Public health	5 (9%)
	Others	4 (7%)
	Strategist	2 (3%)
	Public Health pharmacist	1 (2%)
	Principal public health practitioner	1 (2%)
	Public health academic	1 (2%)
	Allied health practitioner	1 (2%)
Area of Specialty n = 51	General	13 (24%)
	Health Improvement	11 (20%)
	Health Protection	10 (19%)
	Healthcare Public Health	10 (19%)
	Commission	3 (6%)
	Screening	2 (4%)
	Sexual Health	1 (2%)
	Substance abuse	1 (2%)
Gender n = 54	Female	36 (67%)
	Male	16 (30%)
	Prefer not to say	2 (4%)
Location of practice (Country) n = 54	England	32(59%)
	Scotland	15(28%)
	Wales	7(13%)
Location of practice (Region) n = 22	Midlands	7 (22%)
	South West	6 (19%)
	South East	5 (16%)
	North East and Yorkshire	4 (13%)
	North West	4 (13%)
	London	4 (13%)
	National	2(6%)
	East of England	0
Description of main area(s) of work n = 31	Local Authority council	14 (44%)
	Public Health England—regional/local	7 (22%)
	Public Health England—national	2 (6%)
	Acute national health service (NHS) trust	1 (3%)
	Health boards or trusts	1 (3%)
	Clinical Commissioning Group (CCG)	1 (3%)
	University	1 (3%)
	Professional body—regional/local	1 (3%)
	Military	1 (3%)
	Mental health trust	1 (3%)
	Primary care network	1 (3%)

**Table 5:** Benefits, barriers, and placement opportunities for pharmacy professionals in public health teams.

Benefit of having pharmacy professional specialise in public health n = 52	Very Beneficial	Beneficial	Somewhat Beneficial
	26 (50%)	19 (27%)	7 (13%)
Barriers for pharmacy professional to get involved in population health n = 50	Yes	No	Not sure
	30 (60%)	13 (26%)	1 (2%)
Organisation provides placement to funded pharmacy professional for fellowship in public health n = 32	Yes	No	Maybe
	10 (21%)	6 (13%)	16 (34%)
Local Authority inclusion of medicine service as part of MOU with CCG n = 19	Yes	No	Not sure
	1 (3%)	4 (14%)	14 (47%)

**Table 6:** Themes and examples of the recommendation statements from workshop participants on their views of how to steer national changes to improve pharmacy professionals’ contributions to public health across the four UK nations, following the presentation of findings from surveys, a literature review, and the call for evidence.

Themes	Number of Recommendation Statements per Theme	Examples of Individual Recommendation
Public Health Skills and Training	32	*“Develop a career development pathway that does not require pharmacy professionals to work outside the speciality to be recognised as qualified public health professionals”.* *Align with PPH competencies within undergraduate and postgraduate degrees, ranging from:* *i. Undergraduate core fundamental training;* *ii. Postgraduate studies to embed alongside clinical work;* *iii. Specialism in PPH, including joint recognition/registration with GPhC and FPH.* *“Need to look at opportunities presented by reforms to initial education and training of pharmacists to embed training in PPH”.*
National Strategic Approach	31	*“Define national standards for population health knowledge to support consistency across all localities of Great Britain and support capability for roll out of national services”.* *“Contextualise pharmacist practice within health policy so as to understand how the work undertaken improves the health of communities and addresses health inequalities. The commitment and endeavour that this entails needs to be recognised within a career structure that rewards the practitioner with enhancing clinical skills”.* *“Improve strategic engagement with health and care system policy makers/commissioners to influence PH policy and maximise the PPH offer through the whole pharmacy workforce”.*
Commissioning:	13	*“Increase involvement of pharmacy in the commissioning process”.* *“Develop national service specifications for commissioners—to reduce commissioning variation across PH services, drive uniform training and accreditation requirements and reduce cost of service development”.* *“All ICSs should have PPH representation”*
Expanding Service Delivery beyond Community Pharmacy	10	*“Bring elements of public health into pharmacist practice”.* *“Better integrate the sector into the primary care team. The appropriate service(s) should provide the right population interventions, while assisting general practice with case finding and patient/public interventions to improve care and long-term health”.*
Integration of Pharmacy to Better Support PH Protection and Improvement Goals	3	*“Involve pharmacy in leading public health services,* e.g., *National Centre for Smoking Cessation and Training practitioners”.* *“Promote the delivery of PH programmes* via *community pharmacies to improve health and prevent disease, particularly those aimed at primary prevention”.*
Mitigating Health Inequalities:	3	*“Each ICS health inequalities agenda to produce a review of what pharmacy can do to make a difference”.* *“The reach of pharmacies into communities experiencing socioeconomic disadvantage compares very well with that of other providers. Many of the non-communicable diseases exhibit a social gradient, with poorer communities experiencing a greater burden of disease. Access to pharmacies by these communities provides opportunity to improve health and address inequalities”.* *“Addressing health inequalities through focused and integrated public health interventions through pharmacy professionals in areas with high population of underserved communities”.*
Embedding Optimisation of Medicines at a Population Health Level:	1	*“Improve the integration / joint working between ICS/LA/PHE to address use of medicines for population health management—as currently addressed at individual sector level but not at strategic level”.*
Emergency Preparedness, Resilience and Response	1	*“Learn from the COVID-19 pandemic experience, particularly regarding the role of specialist pharmacy services and specialist pharmacists working across PHE, NHSEI and CCG’s and how they should be included as part of EPRR future planning processes”.*

## Data Availability

The data are not publicly available due to privacy or ethical restrictions.

## Supplementary Materials

The following supporting information can be downloaded at: https://www.mdpi.com/article/10.3390/pharmacy13020037/s1, Supplementary Material S1: UK-wide survey for pharmacy professionals with an interest in public population health; Supplementary Material S2: Survey for public health professionals about pharmacy professionals (final); Supplementary Material S3: Call-for-evidence form—PPH manuscript; Supplementary Material S4: SlidoPage: PPH Workshop—contributions from participants; Supplementary Material S5: Recommendations from workshop participants; Supplementary Information S1: Completed Checklist for Reporting Of Survey Studies (CROSS); Box S1—Agenda—PPH Workshop 02 September 2021; Box S2. Examples of quotes on barriers provided by pharmacy technician respondents; Box S3. Examples of opportunities highlighted by pharmacy technicians; Box S4: Knowledge mobilisation of the project and next steps; Table S1: Barrier themes and sample quotes (pharmacy professionals survey); Table S2: Opportunity themes and sample quotes (pharmacy professionals survey); Table S3: Barriers highlighted by public health professionals.
