# Supplementary material for: Pharmaceutical Public Health: A Mixed-Methods Study Exploring Pharmacy Professionals’ Advanced Roles in Public Health, Including the Barriers and Enablers
Source: Pharmacy (Basel). 2025 Mar 1;13(2):37. doi: 10.3390/pharmacy13020037 (PMC11932277; doi:10.3390/pharmacy13020037)
Supplement: Supplementary file 1 [file pharmacy-13-00037-s001.zip › Supplementary S1_UK-Wide Survey for Pharmacy professionals with an interest in public_ population health.pdf]

UK-Wide Survey for Pharmacy professionals with an interest in public/ population health

Dear Colleague,

You are invited to participate in a short survey of pharmacy professionals who have an interest or experience in public health or population health. Please click submit for your responses to be recorded.

This is part of a wider programme of work led by Dr Diane Ashiru-Oredope, commissioned by the four UK Chief Pharmaceutical Officers.

The survey should take approximately 9 minutes to complete. You do not have to provide any identifiable information. Your views are important to us and we take privacy very seriously, so your details will be kept completely confidential.

The data will be used only for the purposes of this survey, reporting on the findings in the pharmaceutical public health review and in further developing recommendations.

The survey has been designed to:

* Explore the number of pharmacy professionals who have experience in leading public/population health projects or have completed/ are undertaking additional public/ population health qualifications,
* Explore the context in which pharmacists are currently involved in public/ population health related roles (excluding nationally commissioned public health services)
* Understand the drivers and barriers associated with pharmacists undertaking public/ population health roles

There is also an opportunity on the final page to connect to a network of pharmacy professionals focused on public/population health.

Thank you for your time and we hope this work will make a difference to our professional practice.

Please do feel free to share this survey link with other colleagues - https://forms.office.com/r/hXqgyNLNZB

If you have any questions, do not hesitate to contact Diane via d.ashiru-oredope@nhs.net

Survey Project Team:
Diane Ashiru-Oredope
Emma McClay
Roeann Osman
Christina Narh

* Required

1. By proceeding to the next page:

   I consent to the information that I provide for the purposes of the survey to be used to inform the Pharmaceutical Public Health Evidence Review and that I do not have to provide any identifiable data/information.

   I have read, understand and agree to the information provided above. *

   ◯ Yes

   ◯ No

2. How do you self-identify? *

- Prefer not to say
- Female (including trans women)
- Male (including trans men)
- Non-binary
- Prefer to self describe

3. Which of the following best describes your ethnic group or background? *

- Prefer not to say
- White - British
- White - Irish
- White - Any other White background
- Mixed - White and Black Caribbean
- Mixed - White and Black African
- Mixed - White and Asian
- Mixed - Any other mixed background
- Asian or Asian British - Indian
- Asian or Asian British - Pakistani
- Asian or Asian British - Any other Asian background
- Black or Black British - Caribbean
- Black or Black British - African
- Black or Black British - Any other Black background
- Other Ethnic Groups - Chinese
- Other Ethnic Groups - Any other ethnic group
- Not stated

4. Which of the following describes your job role? *

- ◯ Pharmacist
- ◯ Pharmacy technician
- ◯ Pre-reg pharmacist
- ◯ Other

5. Which country do you work in? *

- ◯ England
- ◯ Scotland
- ◯ Wales
- ◯ Northern Ireland
- ◯ Other

6. Which region do you work in? *

- ◯ East of England
- ◯ London
- ◯ Midlands
- ◯ North East and Yorkshire
- ◯ North West
- ◯ South East
- ◯ South West
- ◯ National

7. Which of the following describes your main area(s) of work? *

Please select up to a maximum of three roles.

- [ ] Acute national health service (NHS) trust
- [ ] Health boards or trusts
- [ ] Ambulance services
- [ ] Arm's-Length Body/ Organisation such as National Institute for Health and Care Excellence (NICE), Care Quality Commission (CQC), NHS Digital (NOT Public Health England as this is captured seperately)
- [ ] Care home
- [ ] Clinical Commissioning Group (CCG)
- [ ] CCG, with some work into general practices and care homes
- [ ] Commissioning body, national/ regional E.g. NHS England or Health Boards
- [ ] Community Health Services
- [ ] Community pharmacy
- [ ] General practice
- [ ] Health and Justice
- [ ] Sustainability and Transformation Plans (STP) / Integrated Care Providers (ICP) / Integrated Care Systems (ICS)
- [ ] PharmCAS/ 111
- [ ] Private healthcare trust/ service
- [ ] Primary Care Network
- [ ] Mental Health trust
- [ ] Military
- [ ] Local Authority council
- [ ] Public Health England – national
- [ ] Public Health England – regional/ local
- [ ] Professional body – national
- [ ] Professional body– regional/ local
- [ ] University
- [ ] Other

8. Which of the following describes your main area(s) of work? *

Please select up to a maximum of three roles.

- [ ] NHS Ayrshire and Arran
- [ ] NHS Borders
- [ ] NHS Dumfries and Galloway
- [ ] NHS Fife
- [ ] NHS Forth Valley
- [ ] NHS Grampian
- [ ] NHS Greater Glasgow and Clyde
- [ ] NHS Highland
- [ ] NHS Orkney and Shetland
- [ ] General Practice
- [ ] Health Improvement Scotland
- [ ] NHS Education for Scotland
- [ ] NHS National Waiting Times Centre
- [ ] NHS24
- [ ] The State Hospitals Board for Scotland
- [ ] NHS National Services Scotland
- [ ] Military
- [ ] Public Health Scotland
- [ ] Scottish Ambulance Service
- [ ] Community pharmacy
- [ ] Care Home
- [ ] Professional body – national
- [ ] Professional body– regional/ local
- [ ] University
- [ ] Other

9. Which of the following describes your main area(s) of work? *

Please select up to a maximum of three roles.

- [ ] Aneurin Bevan University Health Board
- [ ] Betsi Cadwaladr University Health Board
- [ ] Cardiff and Vale University Health Board
- [ ] Cwm Taf Morgannwg University Health Board
- [ ] Hywel Dda University Health Board
- [ ] Powys Teaching Health Board
- [ ] Swansea Bay University Health Board
- [ ] Welsh Ambulances Services NHS Trust
- [ ] Digital Health and Care Wales
- [ ] General Practice
- [ ] Public Health Wales
- [ ] Health Education and Improvement Wales (HEIW)
- [ ] All Wales Therapeutics and Toxicology Centre (AWTTC)
- [ ] NHS Wales Shared Services Partnership
- [ ] Military
- [ ] Community Pharmacy
- [ ] Care Home
- [ ] Professional body – national
- [ ] Professional body– regional/ local
- [ ] University
- [ ] Other

10. Which of the following describes your main area(s) of work? *

Please select up to a maximum of three roles.

- ☐ Belfast Health and Social Care (HSC) Trust
- ☐ South Eastern HSC Trust
- ☐ Western HSC Trust
- ☐ Southern HSC Trust
- ☐ Northern HSC Trust
- ☐ Powys Teaching Health Board
- ☐ Health and Social Care Board
- ☐ Welsh Ambulances Services NHS Trust
- ☐ Public Health Agency
- ☐ Regulation and Quality Improvement Authority
- ☐ GP Federation Support Unit
- ☐ General Practice
- ☐ Northern Ireland Centre for Pharmacy Learning and Development
- ☐ Medicines Optimisation Innovation Centre
- ☐ Pharmacy Forum NI
- ☐ Military
- ☐ Care Home
- ☐ Community Pharmacy
- ☐ Professional body – national
- ☐ Professional body– regional/ local
- ☐ University
- ☐ Other

Public/Population Health Qualifications

11. How many years have you been qualified? *

12. Do you have any public health training or experience, or a non-formal/ non-university qualification?
For example, a fellowship, a secondment, on-the-job-training

- ◯ Yes
- ◯ No, but I am interested
- ◯ No, not currently a priority
- ◯ Sort of
- ◯ I am not sure

13. Please provide further detail on your training or experience

14. Do you have any formal public/ population health qualifications? *
For example a masters, or certificate.

- ◯ Yes
- ◯ No
- ◯ Other

15. Which public health body are you a member of (full or associate)?

- ☐ Royal Society for Public Health (RSPH)
- ☐ UK Public Health Register (UKPHR)
- ☐ Faculty of Public Health (FPH)
- ☐ Scientist professional registration
- ☐ None
- ☐ Other

16. Which of the following do have the qualifications in or working towards?

	Undergrad	PG Cert/ Dip	Master	MPhil/ PhD	Module	As part of MPH
Public/ population health	◯	◯	◯	◯	◯	◯
Health improvement/ promotion	◯	◯	◯	◯	◯	◯
Health protection	◯	◯	◯	◯	◯	◯
Epidemiology	◯	◯	◯	◯	◯	◯
Infectious and tropical diseases	◯	◯	◯	◯	◯	◯
Health policy	◯	◯	◯	◯	◯	◯
Global health/ global health policy	◯	◯	◯	◯	◯	◯
Health services	◯	◯	◯	◯	◯	◯
Health systems	◯	◯	◯	◯	◯	◯
Other	◯	◯	◯	◯	◯	◯

17. If other, please specify

18. How long have you been using your public health qualification or skills within role(s)? *

- ◯ I have not used my qualification
- ◯ <1 year
- ◯ 1 - 2 years
- ◯ 3 - 4 years
- ◯ 5 + years

19. Which of the following best describes your motivation for undertaking an additional public/ population health qualification(s)? *

Please select no more than three options

- [ ] Ambition to work in public/ population health as a pharmacy professional
- [ ] Ambition to work in public/ population health as an alternative career to pharmacy (i.e Public Health Registrar training or inclusion on the UK Public Health Register)
- [ ] The qualification was required for my role
- [ ] It was recommended to me
- [ ] General interest
- [ ] I don't know
- [ ] Other

20. How much did your pharmacy education (pre-qualification) influence your interest in pursuing a career in public health?

1 = Not at all to 5 = strongly influenced

1	2	3	4	5

Public/Population Work place experience

21. Which of the following public/ population health areas (non-COVID-19 related), best describes the work you are / were involved in or leading on?

Please provide responses for the relevant projects

	Pre COVID-19 pandemic	Since the COVID-19 pandemic	Both pre and since the COVID-19 pandemic
Antimicrobial resistance	◯	◯	◯
Global health	◯	◯	◯
Pharmaceutical Needs Assessment	◯	◯	◯
Health equity	◯	◯	◯
Data analysis/statistics	◯	◯	◯
Commissioning of public health services	◯	◯	◯
Health inequalities/ serving underserved communities	◯	◯	◯
Health improvement	◯	◯	◯
Health protection	◯	◯	◯
Secondary prevention	◯	◯	◯
Disease screening	◯	◯	◯
Long term conditions	◯	◯	◯
Diet and Obesity	◯	◯	◯
Smoking	◯	◯	◯
Alcohol or Substance misuse	◯	◯	◯
Physical activity	◯	◯	◯
Mental Health	◯	◯	◯
Vaccination	◯	◯	◯
Dental hygiene	◯	◯	◯
Wider Determinants of Health	◯	◯	◯

22. Please list any other areas not included above

23. What COVID-19 related public/ population health work are you currently involved in?

24. Have the findings from any of your project(s) (public health or otherwise) been disseminated/ shared wider?

- ☐ No
- ☐ Yes – conference abstract/presentation
- ☐ Yes – peer reviewed publication
- ☐ Yes – reports available online
- ☐ Yes – other publication (e.g. Pharmaceutical Journal or blogs etc.)
- ☐ Yes – guidance, protocols or PGDs

25. Please highlight which of your project(s) that have been disseminated/ shared wider by topic area

	Public health projects	Non-public health projects	Both	None
Conference abstract	○	○	○	○
Conference presentation	○	○	○	○
Publication	○	○	○	○
None	○	○	○	○

26. Please provide the details of your project and how to access the reports/abstracts/presentations

Please provide title and URL to access to project on e.g. website, conference, abstracts, reports etc. Please feel free to use the Call for Evidence webpage to provide further details. Right click and open in another tab - https://forms.gle/cKy2h8zvULmG4u9S7

27. What data sources do you use, if any, to support your work related to public/ population health?

- [ ] PHE Fingertips
- [ ] Office of National Statistics (ONS)
- [ ] Institute for Health Metrics and Evaluation (IHME)
- [ ] Open prescribing
- [ ] National Cancer Registry
- [ ] Hospital Episode Statistics (HES)
- [ ] Local Health
- [ ] Clinical Practice Research Datalink (CPRD)
- [ ] Health inequality dashboard
- [ ] Strategic Health Asset Planning and Evaluation (SHAPE)
- [ ] Other

28. Do you believe there are opportunities for pharmacy professionals to get involved in public/ population health?

- ◯ Yes
- ◯ No
- ◯ Maybe

29. Please explain:

30. Do you believe there are barriers for pharmacy professionals to get involved in public/ population health?

- ◯ Yes
- ◯ No
- ◯ Maybe

31. Please explain:

32. Please list below any work, reports or documents that you are aware of that highlight projects led by pharmacy professionals working or influencing population/public health in UK. Alternatively, please provide details of a colleague we can contact who can provide further details

33. How did you find out about this survey?

- ◯ Colleague
- ◯ Manager
- ◯ Friend
- ◯ Professional body email cascade or newsletter
- ◯ Social media - Twitter
- ◯ Social media - Facebook
- ◯ Social media - LinkedIn
- ◯ Telegram/WhatsApp or other Instant messaging service
- ◯ University
- ◯ Other

34. We would appreciate if you share this survey link with other colleagues. Kindly let us know approximately how many colleagues. If none, please state 0.

35. Thank you very much once again, please use this section to provide any other comments or feedback

36. Would you like to join a network of pharmacists with interest/experience in public/population health? If yes please click on this link to provide details (this is to ensure that your answers are not linked to identifiable data) https://forms.office.com/r/nngn7zCucw. This will open on another tab. Please return to click submit

- ◯ Yes
- ◯ No

This content is neither created nor endorsed by Microsoft. The data you submit will be sent to the form owner.

Microsoft Forms
