# Supplementary material for: Pharmaceutical Public Health: A Mixed-Methods Study Exploring Pharmacy Professionals’ Advanced Roles in Public Health, Including the Barriers and Enablers
Source: Pharmacy (Basel). 2025 Mar 1;13(2):37. doi: 10.3390/pharmacy13020037 (PMC11932277; doi:10.3390/pharmacy13020037)
Supplement: Supplementary file 1 [file pharmacy-13-00037-s001.zip › Supplementary S2_Survey for Public Health Professionals about Pharmacy professionals (Final).pdf]

Survey for Public Health Professionals about Pharmacy professionals (Final)

Dear Public Health Colleague,

There are potentially functions of public health that can benefit from pharmacists' unique expertise including access to care, prevention services as well as pharmacotherapy, pharmacoepidemiology and economics. To better understand this area of work, you are invited to participate in a short survey seeking the views of public health professionals on the contributions of pharmacy professionals* to public/population health (in addition to traditionally/nationally commissioned community pharmacy services) in the four UK nations.

This survey provides an opportunity amongst other things, for public health professionals to share the leadership/operational challenges they face that pharmacy professionals can support with. Your contribution to this survey is really important as it will help in shaping the public health offer across the UK going forward.

Please click submit for your responses to be recorded. We would appreciate responses **before 27 October 2021**

This is part of a wider programme of work, commissioned by the four UK Chief Pharmaceutical Officers.

The survey should take approximately 12 minutes to complete. You do not have to provide identifiable information. Your views are important to us and we take privacy very seriously, so your details will be kept completely confidential.

The findings of this survey along with a call for evidence, literature review and survey for pharmacy professionals with interest in public health will be summarised in publications and included in a report and recommendations to the four UK Chief Pharmaceutical Officers.

The project supports the aims of the NHS Long term plan including the development of new structures of integrated care systems (ICSs) and primary care networks (PCNs) (England); Health and social care delivery plan (Scotland); 'Making Life Better - a whole system framework for public health (2013-23) (Northern Ireland) and A Healthier Wales (Wales) to improve the health and wellbeing of the population of UK by bringing together different professionals including pharmacy professionals to coordinate care better.

The data will be used only for the purposes of this survey, reporting on the findings in the pharmaceutical public health review and in further developing recommendations.

Please do feel free to share this survey link with other public health colleagues - **https://forms.office.com/r/JDVs6Xrcw5**

If you have any questions, do not hesitate to contact Dr Diane Ashiru-Oredope via d.ashiru-oredope@nhs.net or diane.ashiru-oredope@phe.gov.uk

** By Pharmacy Professional, we mean pharmacists or pharmacy technicians*

* Required

1. By proceeding to the next page:

   I consent to the information that I provide for the purposes of the survey to be used to inform the Pharmaceutical Public Health Evidence Review and that I do not have to provide any identifiable data/information.

   I have read, understand and agree to the information provided above. *

   - Yes
   - No

2. Which of the following describes your job role? *

   - Director of Public Health (DPH)
   - Public Health Consultant
   - Consultant in Communicable Disease Control
   - Public Health Specialist
   - Public Health Academic
   - Public Health Registrar ST1-3
   - Public Health Registrar ST 4-5
   - Strategist
   - Practitioner
   - Other

3. Current main area of speciality *

   - General
   - Health Improvement
   - Health Protection
   - Healthcare Public Health
   - Commissioning
   - Screening
   - Other

4. How do you self-identify? *

- Prefer not to say
- Female (including trans women)
- Male (including trans men)
- Non-binary
- Prefer to self describe

5. Which country do you work in? *

- England
- Scotland
- Wales
- Northern Ireland
- Other

6. Which region do you work in? *

- East of England
- London
- Midlands
- North East and Yorkshire
- North West
- South East
- South West
- National

7. Which of the following describes your main area(s) of work? *

Please select up to a maximum of three roles.

- [ ] Acute national health service (NHS) trust
- [ ] Health boards or trusts
- [ ] Ambulance services
- [ ] Arm's-Length Body/ Organisation such as National Institute for Health and Care Excellence (NICE), Care Quality Commission (CQC), NHS Digital (NOT Public Health England as this is captured seperately)
- [ ] Care home
- [ ] Clinical Commissioning Group (CCG)
- [ ] CCG, with some work into general practices and care homes
- [ ] Commissioning body, national/ regional E.g. NHS England or Health Boards
- [ ] Community Health Services
- [ ] Community pharmacy
- [ ] General practice
- [ ] Health and Justice
- [ ] Sustainability and Transformation Plans (STP) / Integrated Care Providers (ICP) / Integrated Care Systems (ICS)
- [ ] PharmCAS/ 111
- [ ] Private healthcare trust/ service
- [ ] Primary Care Network
- [ ] Mental Health trust
- [ ] Military
- [ ] Local Authority council
- [ ] Public Health England – national
- [ ] Public Health England – regional/ local
- [ ] Professional body – national
- [ ] Professional body– regional/ local
- [ ] University
- [ ] Other

8. Which of the following describes your main area(s) of work? *

Please select up to a maximum of three roles.

- [ ] NHS Ayrshire and Arran
- [ ] NHS Borders
- [ ] NHS Dumfries and Galloway
- [ ] NHS Fife
- [ ] NHS Forth Valley
- [ ] NHS Grampian
- [ ] NHS Greater Glasgow and Clyde
- [ ] NHS Highland
- [ ] NHS Orkney and Shetland
- [ ] General Practice
- [ ] Health Improvement Scotland
- [ ] NHS Education for Scotland
- [ ] NHS National Waiting Times Centre
- [ ] NHS24
- [ ] The State Hospitals Board for Scotland
- [ ] NHS National Services Scotland
- [ ] Military
- [ ] Public Health Scotland
- [ ] Scottish Ambulance Service
- [ ] Community pharmacy
- [ ] Care Home
- [ ] Professional body – national
- [ ] Professional body– regional/ local
- [ ] University
- [ ] Other

9. Which of the following describes your main area(s) of work? *

Please select up to a maximum of three roles.

- ☐ Aneurin Bevan University Health Board
- ☐ Betsi Cadwaladr University Health Board
- ☐ Cardiff and Vale University Health Board
- ☐ Cwm Taf Morgannwg University Health Board
- ☐ Hywel Dda University Health Board
- ☐ Powys Teaching Health Board
- ☐ Swansea Bay University Health Board
- ☐ Welsh Ambulances Services NHS Trust
- ☐ Digital Health and Care Wales
- ☐ General Practice
- ☐ Public Health Wales
- ☐ Health Education and Improvement Wales (HEIW)
- ☐ All Wales Therapeutics and Toxicology Centre (AWTTC)
- ☐ NHS Wales Shared Services Partnership
- ☐ Military
- ☐ Community Pharmacy
- ☐ Care Home
- ☐ Professional body – national
- ☐ Professional body– regional/ local
- ☐ University
- ☐ Other

10. Which of the following describes your main area(s) of work? *

Please select up to a maximum of three roles.

- [ ] Belfast Health and Social Care (HSC) Trust
- [ ] South Eastern HSC Trust
- [ ] Western HSC Trust
- [ ] Southern HSC Trust
- [ ] Northern HSC Trust
- [ ] Powys Teaching Health Board
- [ ] Health and Social Care Board
- [ ] Welsh Ambulances Services NHS Trust
- [ ] Public Health Agency
- [ ] Regulation and Quality Improvement Authority
- [ ] GP Federation Support Unit
- [ ] General Practice
- [ ] Northern Ireland Centre for Pharmacy Learning and Development
- [ ] Medicines Optimisation Innovation Centre
- [ ] Pharmacy Forum NI
- [ ] Military
- [ ] Care Home
- [ ] Community Pharmacy
- [ ] Professional body – national
- [ ] Professional body– regional/ local
- [ ] University
- [ ] Other

Pharmacy Professionals and Public/Population Health

11. In current or previous roles, have you ever encountered a pharmacy professional working as a member of any public health team/organisation (Including yourself if applicable)? They may not be working in a pharmacy role *

	Yes	No	Not sure	Not applicable
Your current public health team or public health organisation	◯	◯	◯	◯
Your previous public health team or public health organisation	◯	◯	◯	◯

12. If yes, please describe their role within your **current** public health team/public health organisation

13. If yes, please describe their role within your **previous** public health team/public health organisation

14. Apart from your current or previous roles,

	Yes	No	Don't remember/Dont know	Not applicable
Are you aware of any pharmacy professional who is **also** a public health professional or member of any public health team/public health organisation	◯	◯	◯	◯
Were they employed in their pharmacist/pharmacy technician role	◯	◯	◯	◯

15. Thinking about **12 months pre COVID-19** and within your public health role, did the correct and efficient use of medicines come up as an area of challenge or consideration?

◯ Yes

◯ No

◯ Unsure

◯ Not applicable

16. If applicable, please describe and share examples of **how correct and efficient use of medicines currently or previously come up as an area of challenge or consideration?**

Examples could be using prescription/medicines use data to inform planning and delivery of relevant public health interventions, development of PGDs, providing strategic advice to commission pharmacies to deliver interventions, use of knowledge, intelligence and evidence of treatment effectiveness to inform interventions.

Public/Population Work place experience

17. In your opinion, would there be any benefits of having pharmacists/pharmacy technicians specialising in public health?

- ◯ Little or no benefit
- ◯ Somewhat benecicial
- ◯ Beneficial
- ◯ Very beneificial
- ◯ Unsure
- ◯ Prefer not to answer
- ◯ Other

18. Please explain your response to previous question on benefits of having pharmacy professionals with advanced public health skills

19. If a pharmacist or pharmacy technician were to be funded to join your public health team, please describe how this post could best be utilised?

In addition to your suggestions, please specify (if not already done) if there are any leadership/technical/clinical challenges you face that pharmacy professionals with public health expertise or embedded in public health teams could support with in first place before liaising with pharmacists in other organisations

20. In what areas of population/public health do you feel that there would be benefit achieved by having individuals with pharmacy background working directly as part of your public health team?(tick top 5)

- [ ] Antimicrobial Resistance/Stewardship
- [ ] Cancer
- [ ] Cardiovascular disease
- [ ] Child and maternal health
- [ ] Comparison, practice and performance
- [ ] Dementia
- [ ] Communicable Disease control (excluding pandemics)
- [ ] Climate change
- [ ] Drug Misuse
- [ ] Environmental health
- [ ] Equality analysis
- [ ] Emergency Planning
- [ ] Health Needs Assessments – Health Profiles
- [ ] Health economics and return on investment
- [ ] Health impact assessment
- [ ] Health inequalities
- [ ] Health protection
- [ ] Healthy Living Pharmacy
- [ ] Healthy ageing
- [ ] Injuries and violence
- [ ] Learning disabilities
- [ ] Public Mental health
- [ ] Neurological conditions
- [ ] Obesity, diet and physical activity
- [ ] Oral public health
- [ ] Palliative and end of life care
- [ ] Pandemics
- [ ] Screening
- [ ] Sexual health, reproductive health and HIV

21. Please list any other areas not included above

22. Do you believe there are barriers for pharmacy professionals to get involved in public/ population health?

- Yes
- No
- Other

23. Please explain your reply to question above

24. What level of qualification or experience in public health would you expect a pharmacist wanting to focus on medicines/pharmacy related public health activities to have?

25. Pharmaceutical Needs Assessment (England); Director of pubic health report or delivery planning (Scotland, Wales, Northern Ireland)

	Yes	No	Don't know	Not applicable
England: Has your public health team contributed to a pharmaceutical needs assessment in the last 5 years?	○	○	○	○
England: Did the public health team include a pharmacy professional?	○	○	○	○
Scotland, Wales, NI: Did a pharmacy professional contribute to the latest director of public health report or delivery strategy?	○	○	○	○

26. For England: How involved were the following stakeholders with the development of your last published Pharmaceutical Needs Assessment?

	No at all involved	Somewhat involved	Very involved	Critical to process	Not sure	Not Applicable
Medicines management Team/pharmacy team from the CCG	◯	◯	◯	◯	◯	◯
Local Pharmaceutical Committee (LPC)	◯	◯	◯	◯	◯	◯
NHS England Pharmacy Team	◯	◯	◯	◯	◯	◯

27. England: If you work in a local authority, are medicines/pharmacy services included as part of your memorandum of understanding with the CCG.

◯ Yes

◯ No

◯ Unsure

◯ Not applicable

◯ Other

28. Please list below any work, reports or documents that you are aware of that highlight public or population health projects led by pharmacy professionals working or influencing population/public health in UK. Alternatively, please provide details of a colleague we can contact who can provide further details

29. Would your service/organisation be able to provide a placement to a funded pharmacy professional to complete a secondment or fellowship in public health?

◯ Yes

◯ No

◯ Maybe

◯ Other

30. Which of these terms do believe would best represent pharmacy's contribution to public health

- ◯ Pharmaceutical Public Health
- ◯ Public Health Pharmacy
- ◯ Population Health Pharmacy
- ◯ Other

31. Please add any further comments in relation to your views on a pharmacy professionals' contribution to public health.

32. How did you find out about this survey?

- ◯ Colleague
- ◯ Manager
- ◯ Friend
- ◯ Professional body email cascade or newsletter
- ◯ Social media - Twitter
- ◯ Social media - Facebook
- ◯ Social media - LinkedIn
- ◯ Telegram/WhatsApp or other Instant messaging service
- ◯ University
- ◯ Other

33. Thank you very much once again, please use this section to provide any other comments or feedback

34. If you know of any pharmacists with experience in public/population health, we would be grateful if you can kindly share the link below with them to join a network. https://forms.office.com/r/nngn7zCucw.

◯ Yes

◯ No

35. If you would like to find out more about the project and the findings, please provide your name and email address *

This content is neither created nor endorsed by Microsoft. The data you submit will be sent to the form owner.

Microsoft Forms
