# Supplementary material for: Pharmaceutical Public Health: A Mixed-Methods Study Exploring Pharmacy Professionals’ Advanced Roles in Public Health, Including the Barriers and Enablers
Source: Pharmacy (Basel). 2025 Mar 1;13(2):37. doi: 10.3390/pharmacy13020037 (PMC11932277; doi:10.3390/pharmacy13020037)
Supplement: Supplementary file 1 [file pharmacy-13-00037-s001.zip › Supplementary S3__callforevidenceform_PPH manuscript.pdf]

Appendix V – Call for Evidence

Request for unpublished reports of public health coordinated by pharmacists working at a population/ strategic level in UK

NHS England has commissioned PHAST to carry out a PPH Evidence Review, working with Dr Diane Ashiru-Oredope.

The aim is to identify any documentation describing pharmacists working at a public/population health strategic level in UK. This will support the NHS Long term plan (or equivalent in the devolved domains,) to improve the health and wellbeing of the population of UK by bringing together different professionals including pharmacy to coordinate care better.

We wish to identify any published or unpublished reports or documents or case histories that describe how pharmacists in UK who have received public health training or experience are working at a strategic level and influencing population health in UK. Please only list documents that you can either send as an attachment or send a link to us.

You have been identified as a key stakeholder that could contribute to this review and we would therefore like to invite you to respond to the following short questions.

Please complete the questions listed below and email your responses back to me. Please state NO if you have no information for a question.

Please feel free to forward this email to other colleagues you believe may have information to share.

Your name

Your role:

Your email address:

Name of organisation:

UK country organisation located in:

1. Please list below any reports or documents you are aware of that describe public health trained pharmacists working at a strategic level influencing population health in UK.

-
-
-
-

2. Please list below any reports or documents you are aware of that describe pharmacists involved in expanding public health service delivery beyond community pharmacy and working at a population health level in UK.

-
-
-
-

3. Please list below any reports or documents you are aware of that describe pharmacists working in the field of optimisation of medicines at a population health level in UK.

-
-
-
-

4. Please list below any reports or documents you are aware of that describe pharmacists working in the field of emergency preparedness or emergency response at a population health level in UK.

-
-
-
-

5. Please list below any unpublished reports or documents you are aware of that describe pharmacists working in the field of health protection or health improvement at a population health level in UK.

-
-
-
-

6. Please list below any unpublished reports or documents you are aware of that describe pharmacists working in the field of public health skills training in UK.

-
-
-
-

7. Please list below any unpublished reports or documents you are aware of that describe pharmacists working in the field of health inequalities at a population health level in UK.

-
-
-
-

8. Have you funded staff to undertake PH training in UK? YES / NO

If YES please describe number of staff and type of training.

Would you like to continue supporting this project through a further survey or an interview?   YES / NO

Many thanks for completing this request.
