# Supplementary material for: Pharmaceutical Public Health: A Mixed-Methods Study Exploring Pharmacy Professionals’ Advanced Roles in Public Health, Including the Barriers and Enablers
Source: Pharmacy (Basel). 2025 Mar 1;13(2):37. doi: 10.3390/pharmacy13020037 (PMC11932277; doi:10.3390/pharmacy13020037)
Supplement: Supplementary file 1 [file pharmacy-13-00037-s001.zip › Supplementary S4_PPH Workshop - contribution from participants.pdf]

Do you find Slido easy to use?

My interactions Add

Audience Q&A

10 questions
Open

Polls

What were the key points for you from the information shared

0 votes

Which of these terms resonates best with you?

0 votes

What are your top 3 recommendations? Please add o...

1 vote

What questions or general comments did you have as you...

Help

Survey

Pharmaceutical Public Health Workshop - Contributions from Participants

Open text
23 votes

What were the key points for you from the information shared

Multiple choice
25 votes

Which of these terms resonate best with you?

Open text
11 votes

What other phrase would be better suited

Open text
23 votes

Stop ← Prev Next →

Pharmaceutical Pub...
25 votes

View as participant

Help
