# Supplementary material for: Pharmaceutical Public Health: A Mixed-Methods Study Exploring Pharmacy Professionals’ Advanced Roles in Public Health, Including the Barriers and Enablers
Source: Pharmacy (Basel). 2025 Mar 1;13(2):37. doi: 10.3390/pharmacy13020037 (PMC11932277; doi:10.3390/pharmacy13020037)
Supplement: Supplementary file 1 [file pharmacy-13-00037-s001.zip › Supplementary S5_recommendations_from workshop participants.pdf]

Recommendations by theme

1. National Strategic Approach

a. Define national standards and career pathway
b. Engage health and care system policy makers / commissioners / legislation
c. ICS to identify population health priorities from which pharmacy plans can address use of medicines for population health management
d. Construct an effective national professional development network
e. Continual sharing of good practice models (e.g. capture and measure using QI methodology / tools) and ongoing research
f. Advocate champions for pharmacy professionals in PPH both within and outside the pharmacy profession

- "Improve strategic engagement with health and care system policy makers / commissioners to influence PH policy and maximise the PPH offer through the whole pharmacy workforce"
- "Transform the role of community pharmacists through national policy"
- "Define national standards for population health knowledge to support consistency across all localities of GB and support capability for roll out of national services"
- "Each ICS to identify three local population health priorities (from which pharmacy plans can be developed)"
- "Develop a clear national strategy"
- "Define scope and principles of PPH"
- "Present outputs of this research project to relevant pharmacy and public health bodies in order to educate decision makers and inform future policy around PPH"
- "Contextualise pharmacist practice within health policy so as to understand how the work undertaken improves the health of communities and addresses health inequalities. The commitment and endeavour that this entails needs to be recognised within a career structure that rewards the practitioner with enhancing clinical skills"
- "Drive national and local PH by strategy not simply by a 'nice (thing) to do' to ensure effective contribution from the pharmacy"
- "Advocate for uniformity in local public health services to develop an evidence base for community pharmacy's impact on public health"
- "Improve the integration / joint working between ICS / LA / PHE to address use of medicines for population health management – as currently addressed at individual sector level but not at strategic level"
- "Strengthen the strategic and operational structure to support staff working in PPH. Construct an effective professional development network"
- "Promote systems-level improvements and related policy activity to promote public health pharmacy practice"
- "Define the scope and breadth of PPH principles and practice in the UK"

- “Clearly define the need/expectations/roles associated with PPH”
- “There should be active consultation with Public Health policy makers in government about their vision and strategic plan for delivering the PH ambitions of the NHS Long-Term Plan”
- “Need for shared learning across GB countries”
- “Put in place infrastructure to support and direct the local efforts of pharmacists”
- “Welcome a wider survey of the PH community to provide assurance of support and endorsement of a potential expansion of public health pharmacy”
- “Pharmacy staff should take their audit / research findings to public health conferences and submit to PH journals / newsletter / media”
- “Pharmacy policy makers should engage urgently with government PH policy makers”
- “Include actions to minimise the impact of pharmaceuticals on the environment within PPH strategies”
- “Find champions of PPH outside the pharmacy professions”
- “Develop a compelling narrative for PPH for communicating the need for the specialism and for socialising/communicating with non-pharmacy professionals”
- “Use ambassadors to promote the involvement of pharmacy professionals in public health and the potential value added by their involvement”
- “Future national policies to focus on:
    - Ways to effectively integrate community pharmacy into primary care”
- “Undertake a funding reform to promote greater pharmacy involvement in population health”
- “Consider / address gaps such as strategic leadership for PPH policy / strategy / implementation, surveillance and assessment of population health, emergency response planning”
- “Understand and define the different aspects of public health across the pharmacy services e.g. public health in community pharmacy and pharmacists supporting public health teams in local authorities (or other more strategic roles) - rather than putting them all under the same description”
- “When looking at system level improvements, ensure that data is captured and measured to demonstrate improvements using Quality Improvement (QI) methodology and tools”
- “Amend legislation for Patient Group Direction to include pharmacy tech. This would allow expansion of community led clinics with ~20,000 further healthcare professionals able to add to the PH agenda”

2. Expanding Service Delivery Beyond Community Pharmacy

a. Develop shared vision across pharmacy services
b. Integrate sector within primary care team
c. Engage multidisciplinary team and ICS

- “Future national policies to focus on:
    - Ways to develop a shared vision for different levels of pharmacist services”
- “Better integrate the sector into the primary care team. The appropriate service(s) should provide the right population interventions, while assisting general practice with case finding and patient / public interventions to improve care and long-term health”
- “Consider role of PPH in ICS”
- “Bring elements of public health into pharmacist practice”
- “Recognise the breadth of work being done currently by pharmacists in LAs”
- “Recognise LA pharmacy specialists as part of pharmacy workforce, e.g. workforce survey / plan and put training in place to support junior pharmacists moving into role to allow continuity of organisational/specialist subject memory”
- “Need to ensure we engage on multidisciplinary level rather than focus solely within pharmacy, and within pharmacy engage at an ICS level rather than solely community pharmacy”
- “Each ICS to prepare compulsory ICS driver diagrams selecting top priority for population health pharmacy”
- “Further explore primary care PHH”
- “Consider an MDT approach for Public health planning including pharmacy professionals”

3. Embedding Optimisations of Medicines at a Population Health Level

a. Clarify PPH, to include medicines optimisation

- “Clarify what PPH involves. Clearly optimising medicines is one element and medicines do prevent ill health, not just treat ill health”

4. Emergency Preparedness, Resilience and Response

5. Integration of Pharmacy to Better Support PH Protection & Improvement Goals

a. Promote PPH community pharmacy programmes
b. Involve pharmacy in PH leadership

- “Promote the delivery of PH programmes via community pharmacies to improve health and prevent disease, particularly those aimed at primary prevention”
- “Need for greater integration of community pharmacy within primary care”
- “Involve pharmacy in leading PH services, e.g. NCSCT practitioners”

6. Public Health Skills Training

a. Define PHH career pathway
b. Align to PPH competencies within undergraduate and postgraduate degrees, ranging from:
   i. Undergraduate core fundamental training
   ii. Postgraduate studies to embed alongside clinical work
   iii. Specialism in PHH, including joint recognition/registration with GPhC and FPH
c. Embed PHH into pharmacy technician training
d. Create research opportunities
e. Create in-house training programmes to engage staff with public health, to include health policy, financial drivers of population health
f. Encourage dissemination of research / audits / projects, e.g. PJ involvement

- "Align / define PPH career pathway within the wider career portfolio / frameworks available in and out of pharmacy"
- "Review the undergraduate / postgraduate pharmacist and technician educational offers and identify gaps against PPH competencies"
- "Explore research opportunities"
- "Training for all pharmacy sectors"
- "Education and training in public health services delivered in a pharmacy environment to form part of IET of all pharmacy professionals – consider Miller's triangle approach used by GPhC"
- "Develop pharmaceutical expertise within LAs/ICSs"
- "Share education and knowledge within the profession, e.g. create a campaign / network / forum of pharmacy professionals interested in public health"
- "Incorporate structured public health learning into undergraduate and postgraduate training, highlighting the opportunities that exist (or could exist) for pharmacy professionals. Running alongside that, develop PPH career development pathway or framework to help individuals map competencies"
- "Improve pharmacist training to include PH knowledge, especially health policy and financial drivers of population health to improve population outcomes, and opportunities for professional advancement"
- "Define and measure efforts of effective contribution"
- "Embed PH as a topic in the undergraduate curriculum for pharmacy and in pharmacy technician training to build workforce capability"
- "Consider how to develop and maintain PPH specialists and what the training pathway should look like - link to foundation and advanced pharmacist pathways"
- "Create clear training pathway for pharmacists looking to pursue careers in public health"
- "Increase education and awareness from undergraduate level about the opportunities for pharmacy professionals to pursue a public health career"
- "Improve pharmacist training to include public health knowledge, especially health policy and financial drivers of population health to improve population outcomes as well as improve opportunities for professional advancement"

- “Develop career pathway for pharmacists in PH without the need to give up pharmacy (e.g. joint recognition between Faculty of PH and GPhC)”
- “Develop a career development pathway that does not require pharmacy professionals to work outside the speciality to be recognised as qualified PH professionals”
- “Need to look at opportunities presented by reforms to initial education and training of pharmacists to embed training in PPH”
- “Consider developing career pathways for dedicated PPH roles”
- “Need fundamental training on public health (pre-qualification) to ensure a minimum core set of knowledge and skills to ‘make every contact count’”
- “Consider modules appropriate within wider CPD for pharmacist i.e. MSc courses”
- “Need a balance between specialisation vs imbedding skills in pharmacy workforce”
- “Pharmacy technicians should also be given opportunities to develop as PPH specialists”
- “End the professional bias towards pharmacists. Give equal consideration to the pharmacy technician profession in respect of research and education. Communicate different access to public health courses (not all at degree level)”
- “Require both a skilled healthcare workforce (jobbing pharmacists) and pharmacists embedded in the public health system (or clear collaborative links)”
- “Get more pharmacists registered with such bodies as FPH and UKPHR”
- “Support pharmacy professionals involved in public health to publish and disseminate projects”
- “Disseminate high-quality case studies of pharmacy staff working in PH and their achievements, to inspire colleagues and convince policymakers”
- “Equip pharmacists to specialise into the PH environment”
- “Equip pharmacists working outside PH with skills and awareness of PH”
- “Better support pharmacists working in public health services as most ‘dropped in the deep end’ - not necessarily needing to complete a public health qualification, but to support a better understanding of public health and how pharmacy experts (pharmacists and technicians) can contribute to this maximising on the skills they have to offer”
- “Consider how pharmacy and public health qualifications can be used together rather than choosing one or the other - which appears to be what people are currently doing (i.e. how can the CPD / revalidation be inclusive rather than exclusive to each professional body)”

7. Mitigating Health Inequalities

a. Tackle health inequalities through PH services
b. Involve pharmacy in ICS health inequalities agenda

- “National programmes to embed public health services to tackle local health inequalities”
- “Each ICS health inequalities agenda to produce a review of what pharmacy can do to make a difference”
- “The reach of pharmacies into communities experiencing socioeconomic disadvantage compares very well with that of other providers. Many of the non-communicable diseases exhibit a social gradient, with poorer communities experiencing a greater burden of disease. Access to pharmacies by these communities provides opportunity to improve health and address inequalities”

8. **Other**

8a. Commissioning

a. Give responsibility for commissioning PPH services and funding to the ICS – which should have pharmacy representation
b. Develop national service specifications for commissioners – to reduce commissioning variation across PH services, drive uniform training and accreditation requirements and reduce cost of service development

- "Review the fragmented commissioning system where some services are commissioned by NHSEI (e.g. flu vaccination) and the majority by local authorities. In line with the move towards commissioning moving to statutory ICSs, give ICS the sole responsibility for commissioning pharmacy public health services irrespective of which sector the service is commissioned from so patients are treated holistically and true standards put in place. ICS should also be given the associated funding to undertake such commissioning responsibilities"
- "Increase involvement of pharmacy in the commissioning process"
- "Future national policies to focus on:
  - Devise new incentive mechanisms for improving quality and outcomes"
- "Utilise community pharmacy providers as care providers within an ICS, using the opportunities and skills that complement the efforts of other health care providers. Base pharmacy contracts on the delivery of healthcare and outcomes, not dispensing volume"
- "Identify further key areas in public health that community pharmacy would be best placed to support and influence the commissioning of national services to support this"
- "Reduce the commissioning variation of public health services"
- "Consider how some of the funding that is supplied to local authorities can be better used at a national level to support the commissioning of national public health services as part of the community pharmacy contractual framework"
- "Develop national service specifications for commissioners to use when commissioning local public health services. This will have the benefit of reducing variation, driving uniform training and accreditation requirements and reducing the cost of local service development"
- "There is a need to address this variation in commissioning to focus on the most cost-effective and impactful services"
- -"Need for more national commissioning of public health services to harness the impact community pharmacy can have and reduce the variable commissioning"
- "Seek to use funding from NHS Digital to fund procurement of one IT platform that all commissioners could use when commissioning a public health service through community pharmacy that also enables evaluation and patient surveys"
- "The further development and commissioning of national services needs to provide new funding as the existing community pharmacy global sum will not sustain new services without additional and ongoing funding"
- "Pay attention to where commissioning areas meet / fringe areas, especially if there is large

Summary of Recommendations

National Strategic Approach/ Commissioning

- Clearer national and regional leadership required
- Define national standards and career pathways for pharmacy professionals in public health – competence and accreditation
- Addressing health inequalities through focused and integrated public health interventions through pharmacy professionals in areas with high population of underserved communities
- Agree terminology
- Review of Pharmaceutical Needs Assessment (PNA) regulations to ensure that the emerging role of pharmacists in public health (such as their key role in addressing health inequalities) is highlighted and that data is formally collected on this as part of the PNA process which is undertaken every three years.
- Enhanced PPH Delivery Beyond Community Pharmacy-Key Priorities
- Emergency planning
- Health protection
- Addressing inequalities
- Medicines surveillance / intelligence - to support understanding of intended and unintended consequences (optimisation / ADRs)
- Integration across primary, secondary and tertiary care

Workforce Development

- Define PPH career pathway to allow pharmacy professionals remain within the profession but contribute/lead on public health including at strategic level
- Better align/embed PPH competencies within undergraduate and postgraduate pharmacy degrees, ranging from:
- Undergraduate core fundamental training
- Postgraduate studies to embed alongside clinical and prescribing
- Specialism in PPH e.g., RPS Consultant credentialing and/or joint recognition/registration with GPhC and FPH (or UKPHR)
- Embed PPH into pharmacy technician training and pharmacy support staff
- Increase training available for pharmacy professionals to undertake high quality research including within practice.
- Create/embed available training programmes for pharmacy professionals to undertake public health activities, including health policy, wider determinants of health and financial drivers of population health

Evidence Development/Research

- Promote the sharing of good pharmaceutical public practice models
- Create and embed high quality research and funding for pharmaceutical public health
- Increase the dissemination and adoption of research / audits / project findings
