# Supplementary material for: Pharmaceutical Public Health: A Mixed-Methods Study Exploring Pharmacy Professionals’ Advanced Roles in Public Health, Including the Barriers and Enablers
Source: Pharmacy (Basel). 2025 Mar 1;13(2):37. doi: 10.3390/pharmacy13020037 (PMC11932277; doi:10.3390/pharmacy13020037)
Supplement: Supplementary file 1 [file pharmacy-13-00037-s001.zip › Supplementary_Material_tables and boxes_revisionv2.pdf]

pharmacy

MDPI

Supplementary Material

Supplementary information S1: Completed Checklist for Reporting of Survey Studies (CROSS)

Section/topic	Item	Item description	Reported on page #
**Title and abstract**			
Title and abstract	1a	State the word “survey” along with a commonly used term in title or abstract to introduce the study’s design.	1
	1b	Provide an informative summary in the abstract, covering background, objectives, methods, findings/results, interpretation/discussion, and conclusions.	1
**Introduction**			
Background	2	Provide a background about the rationale of study, what has been previously done, and why this survey is needed.	2-4
Purpose/aim	3	Identify specific purposes, aims, goals, or objectives of the study.	5
**Methods**			
Study design	4	Specify the study design in the methods section with a commonly used term (e.g., cross-sectional or longitudinal).	5 – 7
Data collection methods	5a	Describe the questionnaire (e.g., number of sections, number of questions, number and names of instruments used).	5 - 7
	5b	Describe all questionnaire instruments that were used in the survey to measure particular concepts. Report target population, reported validity and reliability information, scoring/classification procedure, and reference links (if any).	5-7; supplementary materials 1 and 2
	5c	Provide information on pretesting of the questionnaire, if performed (in the article or in an online supplement). Report the method of pretesting, number of times questionnaire was pre-tested, number and demographics of participants used for pretesting, and the level of similarity of demographics between pre-testing participants and sample population.	5-7
	5d	Questionnaire if possible, should be fully provided (in the article, or as appendices or as an online supplement).	Supplementary

	6a	Describe the study population (i.e., background, locations, eligibility criteria for participant inclusion in survey, exclusion criteria).	5-7; supplementary materials 1 and 2
Sample characteristics	6b	Describe the sampling techniques used (e.g., single stage or multistage sampling, simple random sampling, stratified sampling, cluster sampling, convenience sampling). Specify the locations of sample participants whenever clustered sampling was applied.	5-7; supplementary materials 1 and 2
	6c	Provide information on sample size, along with details of sample size calculation.	
	6d	Describe how representative the sample is of the study population (or target population if possible), particularly for population-based surveys.	5-7; supplementary materials 1 and 2
	7a	Provide information on modes of questionnaire administration, including the type and number of contacts, the location where the survey was conducted (e.g., outpatient room or by use of online tools, such as SurveyMonkey).	5-7; supplementary materials 1 and 2
Survey administration	7b	Provide information of survey's time frame, such as periods of recruitment, exposure, and follow-up days.	5-7
	7c	Provide information on the entry process:<br>–>For non-web-based surveys, provide approaches to minimize human error in data entry.<br>–>For web-based surveys, provide approaches to prevent "multiple participation" of participants.	
Study preparation	8	Describe any preparation process before conducting the survey (e.g., interviewers' 4training process, advertising the survey).	5-7
Ethical considerations	9a	Provide information on ethical approval for the survey if obtained, including informed consent, institutional review board [IRB] approval, Helsinki declaration, and good clinical practice [GCP] declaration (as appropriate).	8
	9b	Provide information about survey anonymity and confidentiality and describe what mechanisms were used to protect unauthorized access.	8
Statistical analysis	10a	Describe statistical methods and analytical approach. Report the statistical software that was used for data analysis.	5-6
	10b	Report any modification of variables used in the analysis, along with reference (if available).	NA

	10c	Report details about how missing data was handled. Include rate of missing items, missing data mechanism (i.e., missing completely at random [MCAR], missing at random [MAR] or missing not at random [MNAR]) and methods used to deal with missing data (e.g., multiple imputation).	6
	10d	State how non-response error was addressed.	
	10e	For longitudinal surveys, state how loss to follow-up was addressed.	NA
	10f	Indicate whether any methods such as weighting of items or propensity scores have been used to adjust for non-representativeness of the sample.	NA
	10g	Describe any sensitivity analysis conducted.	NA
**Results**			
Respondent characteristics	11a	Report numbers of individuals at each stage of the study. Consider using a flow diagram, if possible.	8,15
	11b	Provide reasons for non-participation at each stage, if possible.	NA
	11c	Report response rate, present the definition of response rate or the formula used to calculate response rate.	5
	11d	Provide information to define how unique visitors are determined. Report number of unique visitors along with relevant proportions (e.g., view proportion, participation proportion, completion proportion).	5
Descriptive results	12	Provide characteristics of study participants, as well as information on potential confounders and assessed outcomes.	8,15
Main findings	13a	Give unadjusted estimates and, if applicable, confounder-adjusted estimates along with 95% confidence intervals and p-values.	NA
	13b	For multivariable analysis, provide information on the model building process, model fit statistics, and model assumptions (as appropriate).	NA
	13c	Provide details about any sensitivity analysis performed. If there are considerable amount of missing data, report sensitivity analyses comparing the results of complete cases with that of the imputed dataset (if possible).	NA
**Discussion**			
Limitations	14	Discuss the limitations of the study, considering sources of potential biases and imprecisions, such as non-representativeness of sample, study design, important uncontrolled confounders.	22
Interpretations	15	Give a cautious overall interpretation of results, based on potential biases and	22

		imprecisions and suggest areas for future research.	
Generalizability	16	Discuss the external validity of the results.	24
**Other sections**			
Role of funding source	17	State whether any funding organization has had any roles in the survey's design, implementation, and analysis.	27
Conflict of interest	18	Declare any potential conflict of interest.	27
Acknowledgements	19	Provide names of organizations/persons that are acknowledged along with their contribution to the research.	26-27

Supplementary box 1 - Agenda – PPH Workshop 02 September 2021

Agenda – PPH Workshop 02 September 2021

Introduction – put title and interest in chat (10 mins) – 11:00- 11:15

Presentation from the PPH team – (30 mins) (11:15-11:45)

- Literature and call for evidence
- Survey of Pharmacy professionals
- Survey of Public Health Professionals
- Summary of pharmacists working in public health institutions and NE public health pharmacy network
- Key Recommendations

Presentation on behalf of Health Education Enlgand on the Advanced Clinical Practice (in Public Health) programme (10 mins) (11:45-11:55)

Presentation from Royal Pharmaceutical Society (RPS) on Advanced Specialist and Consultant credentialing for Public Health (10 mins) (11:55-12:05)

Presentation from a Director of Public Health: journey from Pharmacy Technician to DPH and why. (10 mins – including Q & A) (12:05-12:15)

Presentation from North East Public Health Pharmacists Network who are employed 1 or 2 days per week by local authorities (10 mins) (12:15-12:25)

5 min break

Focus Group Discussions (30 mins) – 12:30-1:00pm

- Reflections and one recommendation from the group – 1min max
- Comments on recommendations – discuss each recommendation to determine if to adapt/edit, strengthen
- What needs to change within the profession to enable pharmacists to remain as pharmacists as well as public health specialists
- Impact of COVID on public health and how pharmacists have engaged (in addition to vaccination)

Supplementary Table 1: Barrier themes and sample quotes (Pharmacy Professionals Survey)

Themes	Number	Sample quotes
Limited career opportunities/ no defined career pathway	39	• *No clear career pathway, very few boards have pharmacy public health posts.*<br>• *There is a lack of job opportunities for pharmacy professionals within public health teams themselves as there is a lack of recognition of the core knowledge and qualification that pharmacy professionals possess.*<br>• *There is also a lack of clarity with regards to professional management of the pharmacy professional within public health.*<br>• *Not a traditional role. Used to be common placed for a PH pharmacists in boards but sadly no longer the case*
Poor professional recognition	34	• *Not always seen as public health champions*<br>• *the profession is often overlooked as a solution,*<br>• *The barriers for pharmacist to be involved in public and population health are:*<br>• *lack of awareness in public health of what pharmacists can bring to the table.*<br>• *lack of awareness in the pharmacy community of the role that pharmacists can play in public health at a policy and strategy level. I suspect many pharmacists will not be aware of needs assessments other than that for community pharmacies.*<br>• *There appears to not be a good understanding from other healthcare professionals and the general public of the impact pharmacy professionals could have given the opportunity*<br>• *Pharmacist are not seeing as a profession that can contribute to public health*<br>• *"Pharmacy technicians are not on the MHRA list of HCPs who can supply /administer medicines under a PGD. This is a systemic barrier to multiple opportunities to support public health, e.g. administering flu vaccines, continued supply of regular medicines following monitoring, e.g. statins, anti-hypertensives. There are a host of other examples but this is fundamentally a piece of legislation that requires amendment to enable circa 20k*

		professionals to support PH more autonomously within structured safeguards<br>• *Pharmacist IPs could optimise medicines in multiple settings if they were the first option when commissioned services are designed"*
Limited resources (time or financial)	32	• *capacity- pharmacists workload, less protected time for research/QI, under-resourced profession in multiple sectors*<br>• *Busy on daily task. No time to put aside to capture data to understand impact of our daily work on public health.*<br>*No time to design audits.*<br>• *Prohibitive costs associated with studying a Master's course and lack of sponsorships for experienced healthcare professionals from high income countries.*<br>• *capacity, resources and whether it is seen as economically viable*
Lack of training and support	30	• *lack of pharmacy specific formal training that can easily be accessed. to progress in public health as a pharmacist means moving away from being a pharmacist to become a public health specialist/consultant.*<br>• *Pharmacy technicians for example are only required to 'know' about public health issues and not to be able to demonstrate how they can tackle them.*
Inadequate Public Health knowledge	21	• *Lack of understanding of the difference between individual and population health and how inadvertent actions to do better for every individual may actually widen inequality.*<br>• *No undergraduate training in epidemiology and/or data science. Pharmacy degree doesn't set people up very well for research. There's too much focus on completing clinical diploma post-reg for people to consider a career in PH*<br>• *I feel that a lot of pharmacists don't consider aspects of what they are already doing as public health. Having this broader understanding may change the way they think about delivery of certain services and care.*<br>• *Public health not a core part of the pharmacy degree (that I am aware of)*<br>• *Training on Health promotion and changing health behaviours would be helpful for all pharmacists.*

		• *There are vast opportunities for pharmacy professionals to be involved in public health but the initial education of neither profession enables a natural progression towards that.*
Organisational and structural barriers	19	• *workload and staffing structures & wider corporate agenda (large multiples) for Community pharmacy*<br>• *For popoulation health lack of understanding within pharmacy senior leadership in practice settings, lack of education and training in this area (prior experience and access to), no pharmacy network*<br>• *Generally work gets focused on medication management leave less scope for work on wider determinants and other aspects of healthcare public health like screening, Health Impact assessment, health promotion programmes though options are increasing.*<br>*More likely to have a public health component to work rather than have it as a primary focus*
Not capitalizing on available opportunities	12	• *Just about not being aware of what the role entails and having experience in publishing research and drafting proposals/business cases.*<br>• *Not knowing the opportunities available*<br>• *Pharmacy professionals advocating for traditional roles*
Poor representation in public health domains	11	• *Pharmacists are not actively targeted for our experiences to work for PH.*<br>• *There aren't many pharmacists directly employed by LAs - I don't know why this is. Provide a service then take it away and see what happens - back to the "proving one's worth" in a political organisation maybe?*<br>• *In some arenas there are perceptions that all avenues are covered. It's only when pharmacists/technicians become involved that new solutions or alternative ways of working are exposed.*

Supplementary Table 2: Opportunity themes and sample quotes (Pharmacy Professionals Survey)

Themes	Number	Sample quotes
A range of Public Health areas pharmacy professionals can get involved in	44	• *There are many opportunities for community pharmacy professionals to be involved in public health interventions depending on capacity, training and commissioning of services e.g. smoking cessation, sexual health, vaccination, substance misuse services, infection prevention and testing/treating and contributing to pathways for overweight and obesity. There are also opportunities for pharmacists employed by health boards to be involved in population health e.g. prescribing /medicines management initiatives. The All Wales Therapeutics and Toxicology Centre has various working groups in which pharmacists can be involved in strategic medicines management/pharmaceutical public health which in some instances links to other sources of data to provide a broader perspective .*<br>• *Infectious disease screening and treatment in addition/association to the work commonly done by nurses, e.g. TB. Hepatitis CBRNE and disaster preparedness. Pharmacists long overlooked. Their expertise lends itself to this.*<br>• *Mass vaccination programs*<br>• *Work with diseases of global importance, e.g. haemorrhagic fevers*<br>*Polypharmacy and de-prescribing*<br>• *Some local authorities are lacking clinically trained staff. I have found that pharmaceutical expertise embedded within and available to support the local authority public health, and wider LA teams is crucial to enable the appropriate/correct collaboration which is needed for commissioning/transformational change/medicines optimisation. Without this, going forward our community pharmacies may not be considered locally or become recognised as place-based assets to reach their full potential to improve health prevention and chronic disease management as part of the NHS Long Term Plan.*<br>• *Significant need and opportunity for pharmaceutical public health skills to be deployed at system and place level to support commissioning of medicines and pharmacy services*
Qualification, knowledge and skills	26	• *Pharmacy technicians are highly trained, knowledgeable and experienced in providing direct patient care, liaising professionally with other healthcare professionals and are experts in medicines supply and storage*<br>• *A lot of what Pharmacy professionals do in primary care is on a population basis and has an*

		immediate link with public health. For example from producing a local guideline to seeing its implementation in practice affects the health of our population.<br>• *We have a unique perspective on health related to medication. This can be valuable in many different areas*<br>• *Based at the heart of local communities community pharmacy professionals are most likely to see the patient first in respect of public health issues especially when related to self care and yet they are not always the first choice for commissioners. The sector cannot play its role in integrated care if is not included at the right tables. Seats at the right tables need to be made available to pharmacists and pharmacy technicians (as opposed to the contractor) so that both professions can maximise their usefulness in this arena.*
Strategic position in the community	18	• *Very important area - pharmacies are embedded in the heart of our communities, see our population more than any other health professional*<br>• *Pharmacy professionals is widely accessible by the general public and key to deliver any public health messages*<br>• *Community pharmacists in particular have an opportunity to engage with the public on PH issues. General Practice and hospital pharmacists also have opportunities to engage with patients during discussion of medication issues.*<br>• *I believe pharmacists are well placed to be involved in public / population health. They have insights into their local areas and communities. They approach health with a hollistic approach whilst still maintaining the traditional clinical role. Thay are more accessible than most other health care professionals and have greater insight into reasoning for lifestyle choices and behaviours, such as addiction, obesity, etc.*<br>• *As accessible healthcare professionals, we have increasing opportunities to identify risk and take a proactive approach to improving the health of populations and individuals.*<br>• *Pharmacists/ technicians are easily accessible on the high street without an appointment to provide advice/ support/ signposting. Lots of opportunity for brief advice in both community and also primary care pharmacy when undertaking medication optimisation/ medication reviews.*
Recent changing health landscape (health policy e.g. long-term plan)	4	• *The long term plan has increased the opportunity available for pharmacy professionals.*<br>• *There is large overlap between public/ population health and pharmacy practice and pharmacists I think have a particular role in pharmaceutical public health.*
COVID	3	• *My colleagues were directly involved with the covid vaccinations*<br>• *Most definitely- involvement in recent Covid vaccination for example.*<br>• *Pharmacists played a central role in the excellent vaccine rollout in the UK, manufacturing of alcohol rubs, in providing advice on the administration and sourcing of medication to be used in*

		COVID19. We are analytical, excellent communicators, efficient and brilliant decision makers. There are many opportunities for us to demonstrate this at a global, regional and national level.
Good public perception	2	• *The public and healthcare professionals trust our judgement and knowledge, so now is the perfect time to showcase our skills in public/ population health.*<br>• *trusted professional, expert in medicines, access to patients,*

Supplementary Box 2. Examples of quotes on barriers provided by pharmacy technician respondents.

"Pharmacy technicians not utilised enough"

"Pharmacy technicians are not on the MHRA list of HCPs who can supply /administer medicines under a PGD. This is a systemic barrier to multiple opportunities to support public health, e.g. administering flu vaccines, continued supply of regular medicines following monitoring, e.g. statins, anti-hypertensives. There are a host of other examples, but this is fundamentally a piece of legislation that requires amendment to enable circa 20k professionals to support PH more autonomously within structured safeguards"

"Pharmacist IPs could optimise medicines in multiple settings if they were the first option when commissioned services are designed"

"The pharmacy technician role is underused and often not appreciated. Technicians are clinically able to provide more services and advice than 20yrs ago."

"pharmacy technicians constrained by outdated laws that do not allow us to practice to our full potential as registered professionals.

"Normally advertised at degree level, which excludes pharmacy technicians"

Supplementary Box3. Examples of opportunities highlighted by pharmacy technicians.

"Pharmacy technicians are highly trained, knowledgeable and experienced in providing direct patient care, liaising professionally with other healthcare professionals and are experts in medicines supply and storage"

"Pharmacy technicians should be utilised more to allow them to provide services through PGD such as COVID-19 vaccinations"

"I believe the clinical knowledge I have as an experienced pharmacy technician would enhance commissioning of services"

"Pharmacists/ technicians are easily accessible on the high street without an appointment to provide advice/ support/ signposting. Lots of opportunity for brief advice in both community and also primary care pharmacy when undertaking medication optimisation/ medication reviews."

Supplementary Table 3: Barriers highlighted by public health professionals

Themes	Number	Sample quotes
Organisational and structural barriers	13	• *"time, staff turnover, recovery from COVID pandemic"*<br>• *"Reluctance to change status quo from senior management and policy down to front-line. Fear of additional workloads in already stretched services (although social prescription and signposting goal would be to reduce reliance), lack of undertanding (not enough data locally or nationally) on the long-term benefits of an increased focus and increase in funding towards social prescription."*<br>• *It seems form my experience of working with pharmacies that they are quite pressured for time and there is a high turn over of counter staff sometime as well as a turn over of commercial owners"*<br>• *gaining of agreement for pharmacists to be willing to do more public health focused work as it could be seem as detracting from their 'core business'.*
Limited resources (time and/or financial)	11	• *Cant think of any specific barriers although availability of pharmacists in the SW is already a challenge and exacerbating that would be a concern*<br>• *capacity, resources and whether it is seen as economically viable*<br>• *Unlikely to be a full time rule so hard to find match an interested person to a small number of hours e.g. 1 day a week.*<br>• *Entry level of public health professional does not support pharmacy pay grades*
Poor professional recognition	10	• *..there is a lack of recognition of the core knowledge and qualification that pharmacy professionals possess.*<br>• *I think we don't always understand each other's areas of work and the 'business' side of pharmacy means certain work takes preference , like primary care.*<br>• *Lack of understanding of wider benefits to self, profession, community and other HC professionals*
Limited career opportunities/ no defined career pathway	9	• *, not many role models or possibly job opportunities, may need to carve out a niche for themselves*<br>• *potentially difficult to maintain professional practice whilst working in PH*<br>• *there is no defined formal route into public health. Some pharmacists can go via Specialty Registrar, and others have used the Defined specialist route (recently refined). The public health role and opportunity for pharmacists need to be integrated into Learning and Academic organisations to get early buy in*<br>• *There is a lack of job opportunities for pharmacy professionals within public health teams themselves as there is a lack of recognition of the core knowledge and qualification that pharmacy professionals possess. Pharmacy professionals may also lack clarity/confidence in moving to a new area of work especially if they feel that they will need to undertake a*

		new qualification to enable them to work within public health. There is also a lack of clarity with regards to professional management of the pharmacy professional within public health.<br>• *Not a traditional role. Used to be common placed for a PH pharmacists in boards but sadly no longer the case*
Lack of training and support	6	• *There will be barriers including limitations to what pharmacists are able to do in their working day, what training they would need to undertake public health work,*<br>• *To be blunt I have been working in XXXXXX for x years and nobody ever suggested I take formal training in this area. People work in silos and as long as you are ticking the boxes, they leave you alone. I feel any senior professional joining a public health organisation without public health training needs to obtain it, fast!*
Not capitalising on available opportunities	4	• *The importance of public health implications of medicines largely unexplored*<br>• *Expectations around what can be achieved with medicines are often limited to cost savings in commissioning. Wider work on reducing medication, working with community teams to ensure medication cocktails are well suited to patients will be more important as more co-morbidities in population. This work isn't generally considered public health but it is- making sure system works together is very important.*
Poor representation in PH domains	3	• *Lack of understanding of wider benefits to self, profession, community and other HC professionals*<br>• *I think we don't always understand each other's areas of work and the 'business' side of pharmacy means certain work takes preference , like primary care.*
Inadequate PH knowledge	2	• *In general our pharmacy colleagues are not PH trained. They are therefore clearly expert in medicines issues, but don't have wider skills in population health approaches and epidemiology. Collaborative working with PH specialists and others overcomes this to a large extent, but some training in PH for at least some of our pharmacists would be helpful.*<br>• *Not covered extensively at undergraduate level. Role of pharmacists in public health very variable and topic specific. Pharmaceutical public health not seem by PH fraternity or professional body as a discipline. No formal training to skill pharmacists up in this area broadly. Community pharmacy contract not remunerated for this work*

Supplementary box 4: Knowledge mobilisation of the project and next steps

Public Health Community: Faculty of Public Health and People in Public Health and National Pharmacy Groups

Key findings of the evidence review have been presented to the four committees of the Faculty of Public Health - Health Improvement, Academic research, health services and health protection as well as the national UK People in Public Health Group, national pharmacy groups led by the Chief Pharmaceutical Officers of England and Northern Ireland.

Conferences:

- *Clinical Pharmacy Congress (May 2022) – oral presentation - Evidence Review of Pharmaceutical Public Health in the United Kingdom*
- *RPS Conference (November 2022) – poster presentation - Barriers and facilitators to pharmacy professionals' specialist public health roles: a mixed methods UK-wide Pharmaceutical Public Health evidence review*
- *FIP Research conference (June 2023): Poster presentation - Rapid evidence review of pharmaceutical public health in high and upper middle-income countries: case studies of mesoand macro level activities*
- *UK Public Health Science conference (November 2023): Poster presentation - Public health qualifications, motivation, and experience of pharmacy professionals: exploratory cross-sectional surveys of pharmacy and public health professionals*
- *UKHSA Conference (November 2023): e-Poster presentation - Specialist contribution of pharmacy professionals to public health in high and upper middle-income countries*
