# Supplementary figures and images for: Pharmaceutical Public Health: A Mixed-Methods Study Exploring Pharmacy Professionals’ Advanced Roles in Public Health, Including the Barriers and Enablers
Source: Pharmacy (Basel). 2025 Mar 1;13(2):37. doi: 10.3390/pharmacy13020037 (PMC11932277; doi:10.3390/pharmacy13020037)

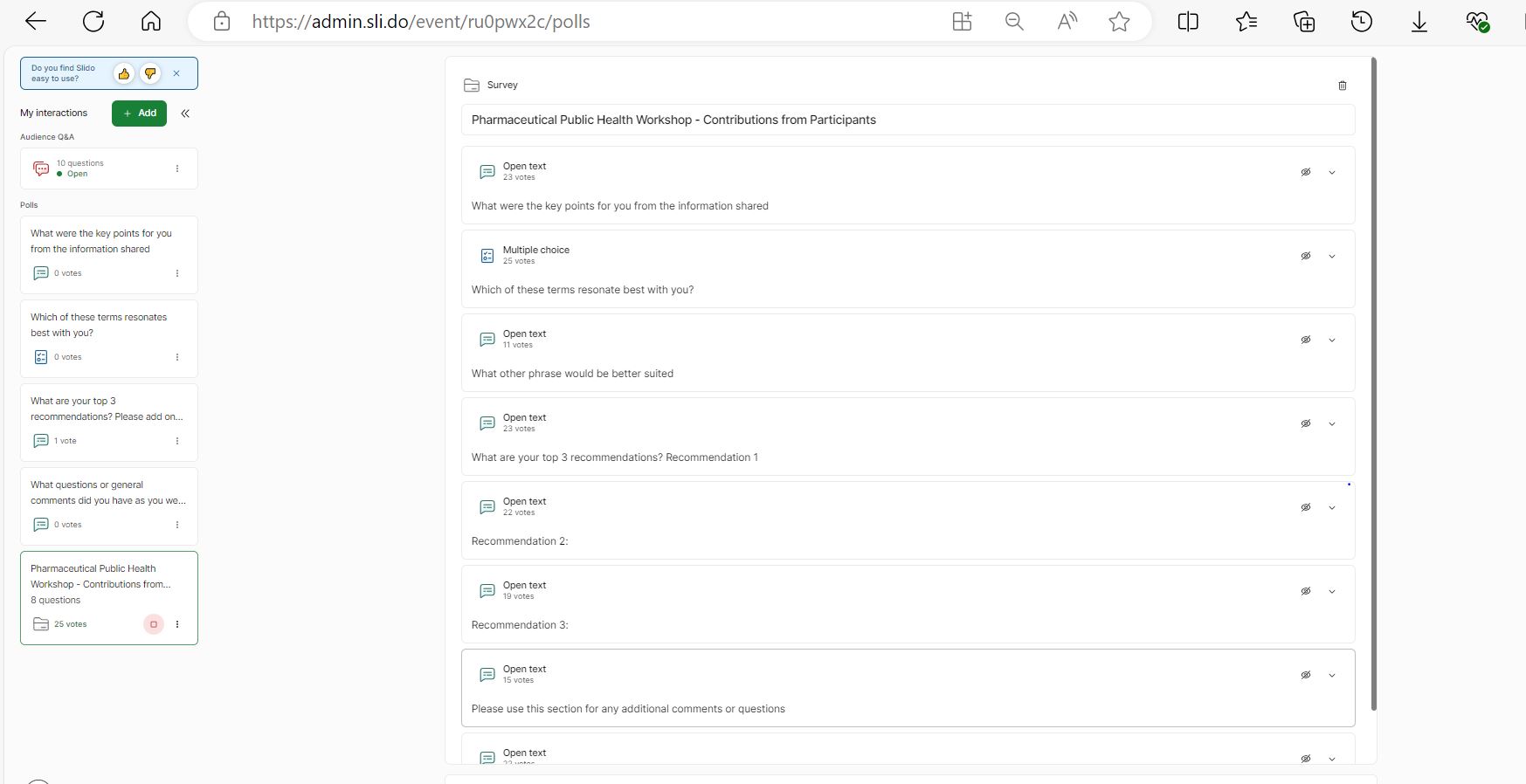

Supplement: Supplementary file 1 [file pharmacy-13-00037-s001.zip › Supplementary S4_SlidoPage_PPH Workshop - contribution from participants.JPG]
